# Supplementary material for: A chemical biology toolbox to study protein methyltransferases and epigenetic signaling
Source: Nat Commun. 2019 Jan 3;10:19. doi: 10.1038/s41467-018-07905-4 (PMC6318333; doi:10.1038/s41467-018-07905-4)
Supplement: Supplementary file 1 — Supplementary Information [file 41467_2018_7905_MOESM1_ESM.pdf]

A Chemical Biology Toolbox to Study Protein Methyltransferases and Epigenetic Signaling

Supplementary Tables, Figures, and Methods

Sebastian Scheer[1], Suzanne Ackloo[2], Tiago S. Medina[3], Matthieu Schapira[2,4], Fengling Li[2], Jennifer A. Ward[5,6], Andrew M. Lewis[5,6], Jeffrey P. Northrop[1], Paul L. Richardson[7], H. Ümit Kaniskan[8], Yudao Shen[8], Jing Liu[8], David Smil[2], David McLeod[12], Carlos A. Zepeda-Velazquez[12], Minkui Luo[9,10], Jian Jin[8], Dalia Barsyte-Lovejoy[2], Kilian V. M. Huber[5,6], Daniel D. De Carvalho[3,11], Masoud Vedadi[2,4], Colby Zaph[1]*, Peter J. Brown[2]*, Cheryl H. Arrowsmith[2,3,11,13]*

[1]Infection and Immunity Program, Monash Biomedicine Discovery Institute, Department of Biochemistry and Molecular Biology, Monash University, Clayton VIC 3800, Australia.
[2]Structural Genomics Consortium, University of Toronto, Toronto ON M5G 1L7, Canada.
[3]Princess Margaret Cancer Centre, University Health Network, Toronto ON M5G 2M9, Canada.
[4]Department of Pharmacology and Toxicology, University of Toronto, Toronto ON M5S 1A8, Canada.
[5]Structural Genomics Consortium, University of Oxford, Oxford OX3 7DQ, UK.
[6] Target Discovery Institute, Nuffield Department of Medicine, University of Oxford, Oxford OX3 7FZ, UK.
[7]AbbVie Inc., 1 North Waukegan Rd., North Chicago, IL 60064, USA
[8]Mount Sinai Center for Therapeutics Discovery, Departments of Pharmacological Sciences and Oncological Sciences, Tisch Cancer Institute, Icahn School of Medicine at Mount Sinai, New York, NY 10029, USA
[9]Chemical Biology Program, Memorial Sloan Kettering Cancer Center, New York, NY 10065, USA
[10]Program of Pharmacology, Weill Cornell Medical College of Cornell University, New York, NY 10021, USA
[11]Department of Medical Biophysics, University of Toronto, Toronto ON M5G 1L7, Canada.
[12]Ontario Institute for Cancer Research, Toronto ON, M5G 0A3, Canada.
[13]Lead Contact

Co-corresponding authors: CA (cheryl.arrowsmith@uhnresearch.ca); PJB (peterj.brown@utoronto.ca); CZ (colby.zaph@monash.edu)

Supplementary Table 1. A summary of the number of off-target proteins screened and the number of 'hits' with *in vitro* Kd values less than 1 μM. The panel includes methyltransferases, ion channels (Eurofins (https://www.eurofinsdiscoveryservices.com) and PDSP (https://pdspdb.unc.edu/pdspWeb/)), kinases (Eurofins). Related to **Fig. 1c**.

Probes	Controls	PDSP, Eurofins	Methyltransferase panel at the SGC	Total off-targets screened	Hits with Kd ≤ 1 μM	Off-targets Eurofins, PDSP[b]	*in vivo* compatible[c]
UNC0642		NT	34	34	0	NT	Y[1]
A-366		NT	34	34	0	NT	Y[2]
A-395*		98	33	139	11	5-HT2B, Alpha-1A, -1B, -1D, -2A, -2B, -2C, H3, NET, SERT, Sigma 2	Y[3]
	A-395N*	95	34	137	7	Alpha-2A, -2B, -2C, H3, NET, SERT, Sigma 1	
GSK343		NT	33	33	0	NA	N
UNC1999		NT	33	33	0	NA	Y[4]
	UNC2400	NT	34	34	0	NA	
OICR-9429*		87	34	121	2	5-HT2B, H3	
	OICR-0547*	87	34	121	0	NA	
BAY-598		78	34	112	2	NK2, A3 (h)	Y[5]
	BAY-369	NT	34	34	0	NA	
(R)-PFI-2		118	34	152	0	NA	N
	(S)-PFI-2	NT	34	34	0	NA	
A-196		180	34	214	3	A1(h), A2A(h), PBR	
	A-197	118	34	152	0	NA	
	SGC2043	NT	34	34	0	NA	
MS023		137	34	171	1	Sigma 1	Y[#]
	MS094	55	34	89	0	NA	
SGC707		140	34	174	0	NA	
	XY1	95	34	129	0	NA	
MS049		143	34	177	3	H3, Sigma1, Sigma 2	
	MS049N	NT	34	34	0	NA	
TP-064		53	34	87	0	NA	
	TP-064N	51	34	85	0	NA	
SGC0946[a]		130	34	164	7	5-HT transporter (h), 5-HT2A (h), 5-HT2C, alpha 1B (h), alpha 2C (h), Na+ channel (site 2)	Y[6]
	SGC0649	NT	34	34	0	NA	
GSK591		135	34	169	8	5-HT1A, 5-HT2B, 5-HT6, Alpha-2A, -2B, -2C, NET, Sigma 1	N
	SGC2096	NT	34	34	0	NA	
LLY-283		53	34	87	0	NA	Y[7]
	LLY-284	51	34	85	0	NA	
SGC3027		NT	34	NT	NT	NT	
	SGC3027N	NT	34	NT	NT	NT	
BAY-6035		NT	34	NT	NT	NT	
	BAY-444	NT	34	NT	NT	NT	

*These targets have also been screened against 8 methyl lysine binding domains and no activity was measured.
[#]IP mouse pharmacokinetic studies, significant plasma concentrations (> 1 μM) of MS023 were achieved by both intraperitoneal (IP) injection and oral administration (Jin lab unpublished results). Thus, MS023 is useful for *in vivo* efficacy studies.

[a] Methyltransferase off-target activity on PRMT4 (IC_{50} = 500 nM)
[b] Eurofins and PDSP screens comprise binding assays only and may not translate into functional activities.
[c] Y means the probe has been used in an *in vivo* study as per reference. N indicates PK/PD properties are not *in vivo* compatible.
NT – not tested; 'NA' – not applicable

Supplementary Table 2. Chemical structures of probes, their chemotype-matched controls, and chemical biology reagents (or optimal site of derivatization). The full structure for the probe or control is the 'Core' plus the moiety in the 'Probe' or 'Control' column, respectively. The point of attachment of the moiety on the core is demarcated by the 'R' within a square. The point of attachment to the moiety in 'Probe' and 'Control' columns is the dashed line. Related to **Figs. 1b and 1c**.

Protein	Core	Probe	Control	Reagents for chemical biology
DOT1L	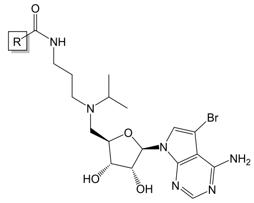	SGC0946 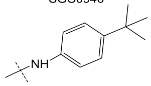	SGC0649 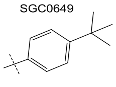	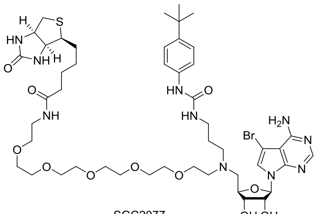 SGC2077
EED	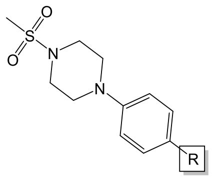	A-395 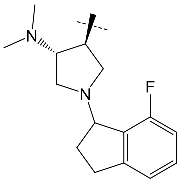	A-395N 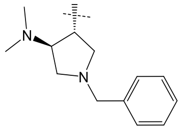	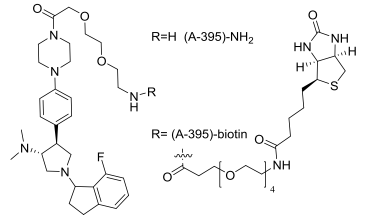 The chemical structure for (A-395N)-biotin is shown in the synthetic methods.
EZH2/H1[4]	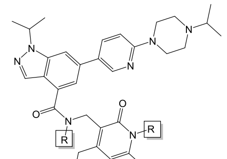	UNC1999<br>H	UNC2400<br>CH_3	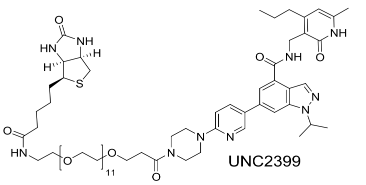 UNC2399
EZH2	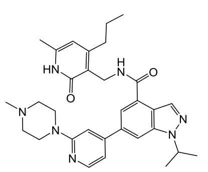	GSK343		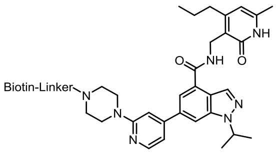 Suggested by SAR
G9a/GLP	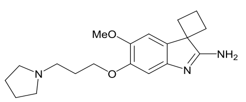	A-366		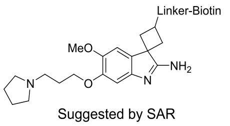 Suggested by SAR
G9a/GLP	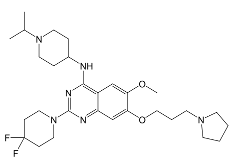	UNC0642		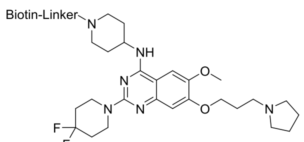 Suggested by SAR

PRMT type I	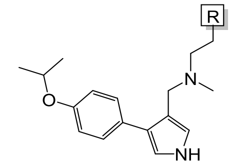	MS023<br>—NH_2	MS094<br>—OH	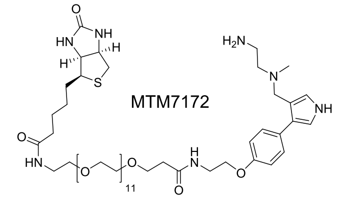
PRMT3	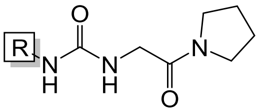	SGC707<br>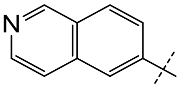	XY1<br>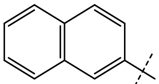	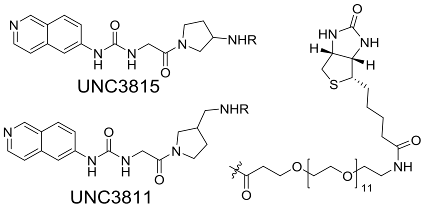
PRMT4	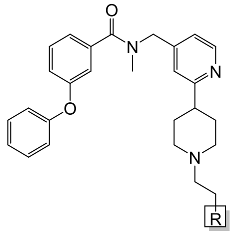	TP-064<br>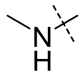	TP-064N<br>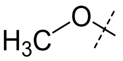	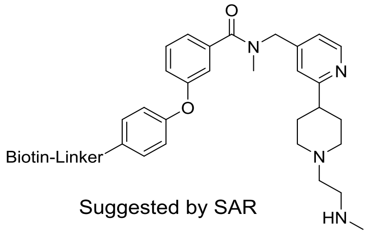
PRMT4	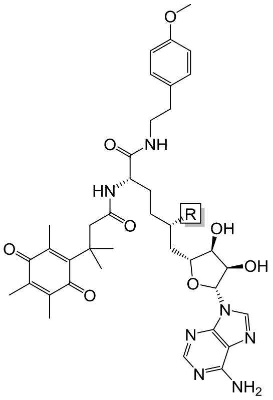	SKI-73<br>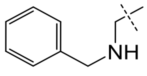	SKI-73N<br>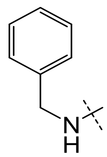	
PRMT4/6	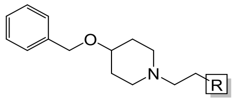	MS049<br>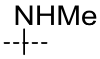	MS049N<br>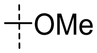	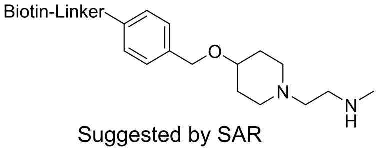<br>Suggested by SAR
PRMT5	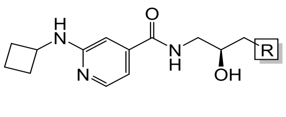	GSK591<br>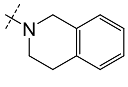	SGC2096<br>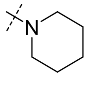	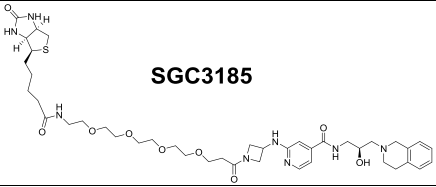
PRMT5	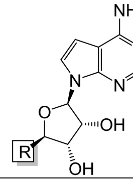	LLY-283<br>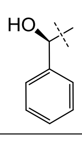	LLY-284<br>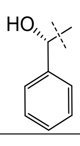	

PRMT7	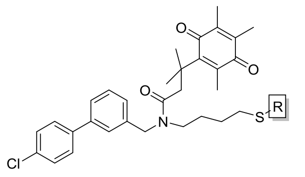	SGC3027 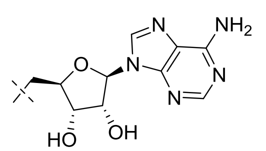	SGC3027N 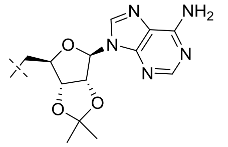	
SETD7	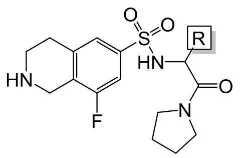	(R)-PFI-2 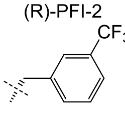	(S)-PFI-2 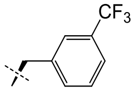	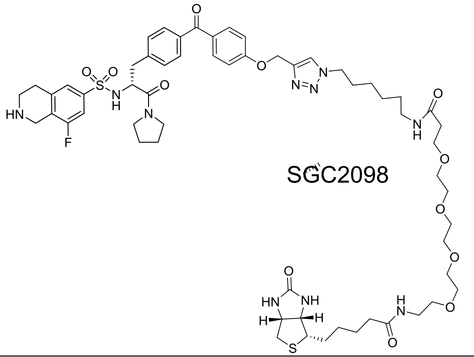 SGC2098
SMYD2	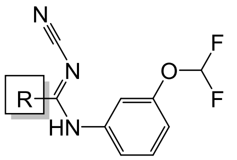	BAY-598 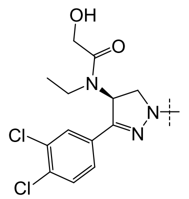	BAY-369 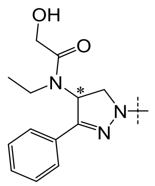	
SMYD3	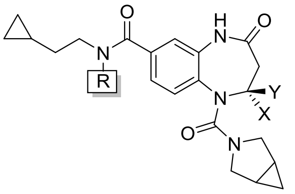	BAY-6035<br>R=H X=Me Y=H	BAY-444<br>R=Me X=H Y=Me	
SUV420H1/H2	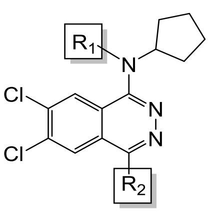	A-196<br>R_1 = H<br>R_2 = 4-pyridyl	SGC2043<br>R_1 = Me R_2 = 4-pyridyl<br><br>A-197<br>R_1=H R_2= 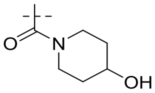	
WDR5	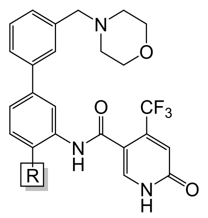	OICR-9429 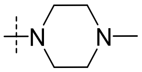	OICR-0547 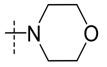	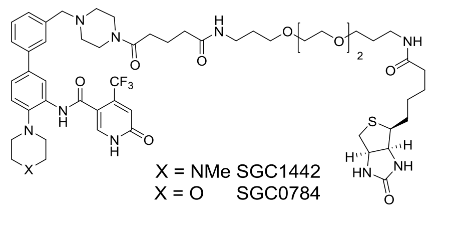 X = NMe SGC1442<br>X = O SGC0784

Supplementary Table 3. Detailed conditions used in the methyltransferase activity assays. Related to **Fig. 1c** and Methyltransferase selectivity assays in METHODS.

Protein		MT (nM)	Substrate	Concentration (μM) Substrate	Concentration (μM) SAM	Buffer (20 mM, pH 8)	DTT (mM)	TCEP (mM)
G9a		5	Biot-H3 (1-25)	1	10	potassium phosphate	0	0
GLP		5	Biot-H3 (1-25)	1	10	potassium phosphate	0	0
SUV39H1		10	Biot-H3 (1-25)	0.2	5	Tris-HCl	5	0
SUV39H2		10	Biot-H3 (1-25)	1	2	Tris-HCl	5	0
SETDB1		2	Biot-H3 (1-25)	0.1	5	Tris-HCl	5	0
PRMT1		15	Biot-H4 (1-24)	0.13	5	Tris-HCl	5	0
PRMT3		20	Biot-H4 (1-24)	1	28	Tris-HCl	5	0
PRMT4		75	Biot-H3 (1-25)	1	2	Tris-HCl	5	0
PRMT5-MEP50 Complex	PRMT5	15	Biot-H4 (1-24)	0.12	1	Tris-HCl	5	0
	MEP50							
PRMT6		50	Biot-H4 (1-24)	1	2	Tris-HCl	5	0
PRMT7		25	Biot-H2B (23-37)	0.3	1	Tris-HCl	5	0
PRMT8		20	Biot-H4 (1-24)	1	2	Tris-HCl	5	0
PRMT9		10	Biot-SAP145 (490-529)	0.08	20	Tris-HCl	5	0
SETD8		50	Biot-H4 (1-24)	20	30	Tris-HCl	5	0
SUV420H1		100	Biot-H4K20me1	3	10	Tris-HCl	5	0
SUV420H2		500	Biot-H4K20me1	1	10	Tris-HCl	5	0
SMYD2		30	Biot-p53 (361-380)	3	0.5	Tris-HCl	5	0
SMYD3		10	Biot-MAP3K2 [(249-273)	15	0.5	Tris-HCl	5	0
BCDIN3D		50	Biot-microRNA-145 (23bp)	0.2	2	Tris-HCl	5	0
DNMT1		100	Biot-Hemimethylated ds-DNA	0.6	2	Tris-HCl	5	0
DNMT3A/3L		10	poly(dI-dC)	0.2	0.3	Tris-HCl	5	0
DNMT3B/3L		20	poly(dI-dC)	0.3	1	Tris-HCl	5	0
NSD1		20	Chicken Nucleosume	0.2	2	Tris-HCl	0	2
NSD2		50	Chicken Nucleosume	0.4	3	Tris-HCl	0	2
NSD3		50	Chicken Nucleosume	0.3	3	Tris-HCl	0	2
ASH1L		100	Chicken Nucleosume	0.3	3	Tris-HCl	0	2
SETD2		150	Biot-H3 (21-44)	1	5	Tris-HCl	5	0
DOT1L		10	Chicken Nucleosume	1	1	Tris-HCl	5	0
PRC2 Complex-EZH1	EED	10	Biot-H3 (21-44)	1	4	Tris-HCl	5	0
	EZH1							
	SUZ12							
	RBBP4							
	AEBP2							
PRC2 Complex-EZH2	EED	20	Biot-H3 (21-44)	1	1	Tris-HCl	5	0
	EZH2							
	SUZ12							
PRDM9		1	Biot-H3 (1-25)	1	60	Tris-HCl	5	0
SETD7		10	Biot-H3 (1-25)	1	1	Tris-HCl	5	0
MLL1 Complex	MLL1	20	Biot-H3 (1-25)	2	2	Tris-HCl	5	0
	RBBP5							
	WDR5							
MLL3 Complex	MLL3	100	Biot-H3 (1-25)	12	55	Tris-HCl	5	0
	ASH2L							
	RBBP5							
	WDR5							
	DPY30							

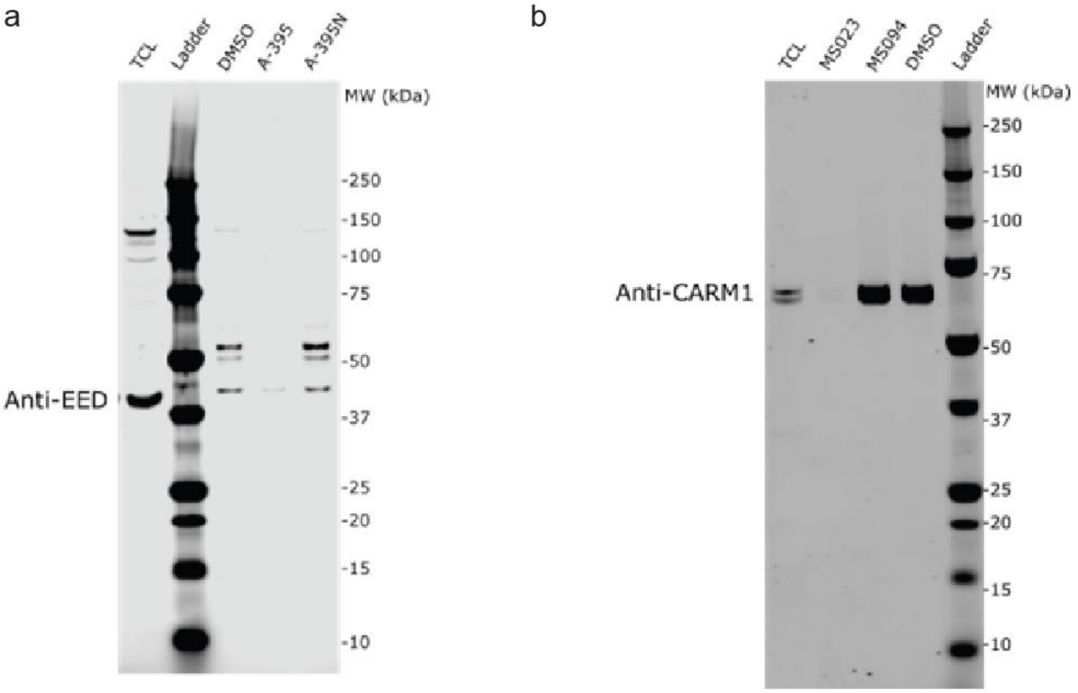

Supplementary Figure 1. Western blot analysis of cognate target engagement by chemoproteomic affinity probes. (a) Western blot analysis of EED (three isoforms of MW = 45, 50 and 53 kDa) enrichment by (A-395)-NH_2 from G401 cell lysate, and competition by pre-treatment with A-395, A-395N, or DMSO control (all 20 µM); TCL = total cell lysate. (b) Western blot analysis of PRMT4 (MW = 70 kDa) enrichment by MTM7172 (whose structure is shown in Supplementary Table 2) from HEK293 cell lysate, and competition by pre-treatment with MS023, MS094 or DMSO control; TCL = total cell lysate. Related to Fig. 3.

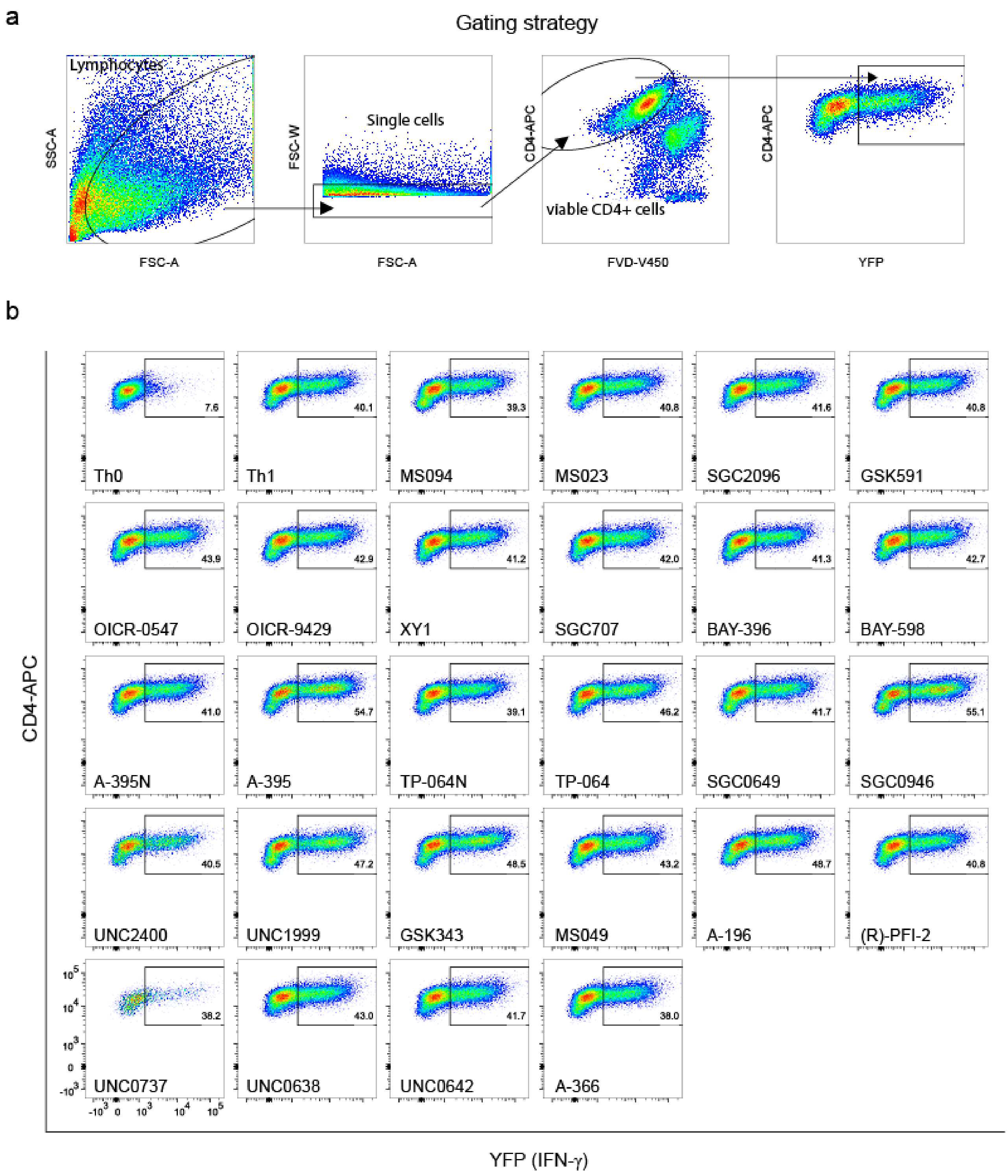

Supplementary Figure 2. FACS analysis of Th1 polarized cells in the presence of indicated compounds. (a) Gating strategy to determine the frequency of YFP+ cells (IFN-γ+) in the population of single and viable CD4+ cells from cultured CD4+ T cells of IFN-γ-YFP reporter mice. (b) Naive CD4+ T cells of IFN-γ-YFP reporter mice were cultured for 4 days under Th0 or Th1 cell polarizing conditions in the presence or absence (Th0, Th1) of indicated compounds (1 μM) and analysed by FACS. x-axis: YFP reporter for IFN-γ, y-axis: CD4. Related to Figs. 4a and 4b.

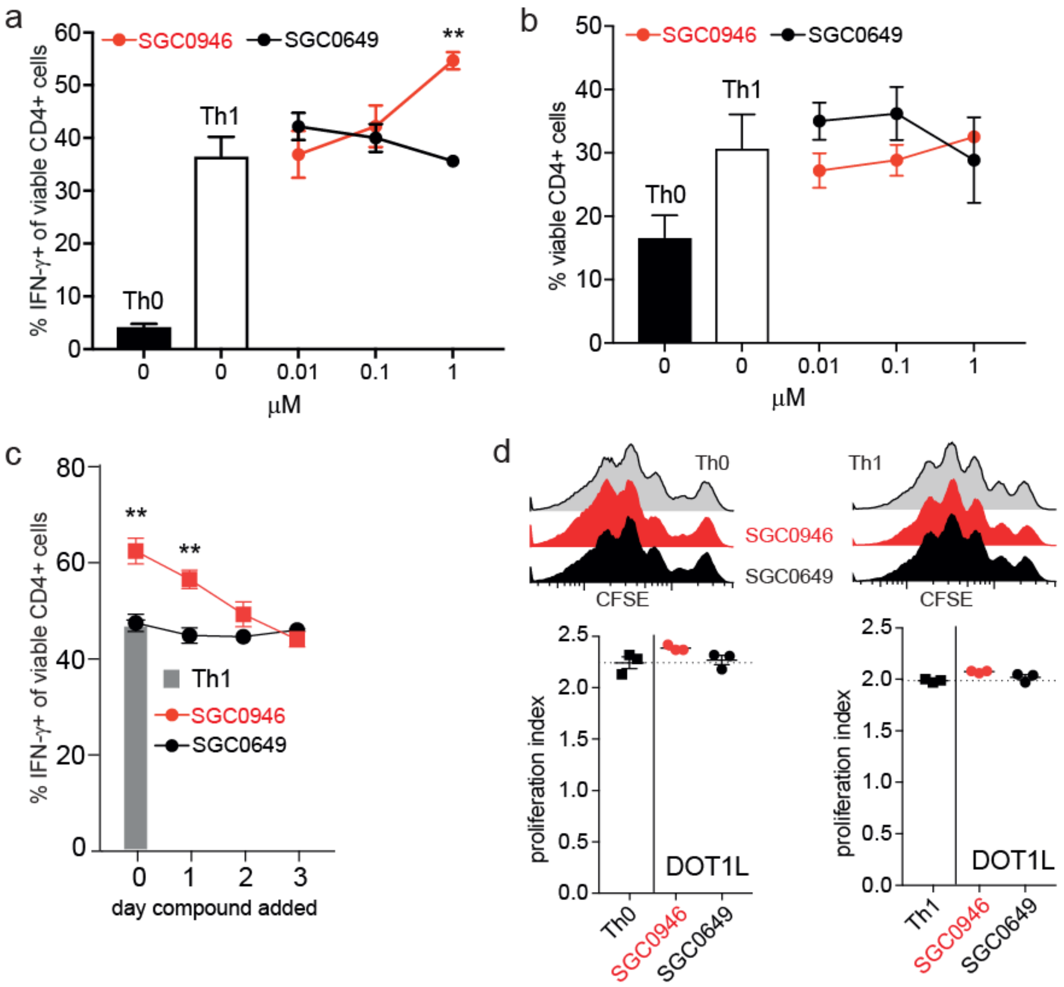

Supplementary Figure 3 Dose- and temporal response analyses of the DOT1L chemical probe (SGC0946) and control (SGC0649) in the context of IFN-γ production, cell viability and proliferation in mice. (a,b) Dose-response analysis for CD4+ T cells cultured for 4 days under Th0 or Th1 cell polarizing conditions in the absence (Th0, Th1) or presence of SGC0649 (Th1) or SGC0946 (Th1) at indicated concentrations regarding (a) frequency or (b) viability of IFN-γ+ CD4+ T cells. Data represents two independent experiments. (c) Time dependency of inhibition by the chemical probe (SGC0946) and control (SGC0649) under Th1 polarizing conditions. The probes were added to the culture at indicated time points and analyzed at day 4. Data represents two independent experiments. (d) Proliferation of CD4+ T cells under Th1 polarizing conditions for 3 days in the absence (Th0, Th1) or presence of SGC0649 or SGC0946 at 1 μM. The proliferation was assessed by CFSE stain by flow cytometry. The proliferation index in (d) was calculated using FlowJo (v10). Data represents three independent experiments. Statistical significance between conditions was determined using one-way ANOVA (**$p \leq 0.01$). Error bars represent SEM. Related to Figs. 4 and 5**.**

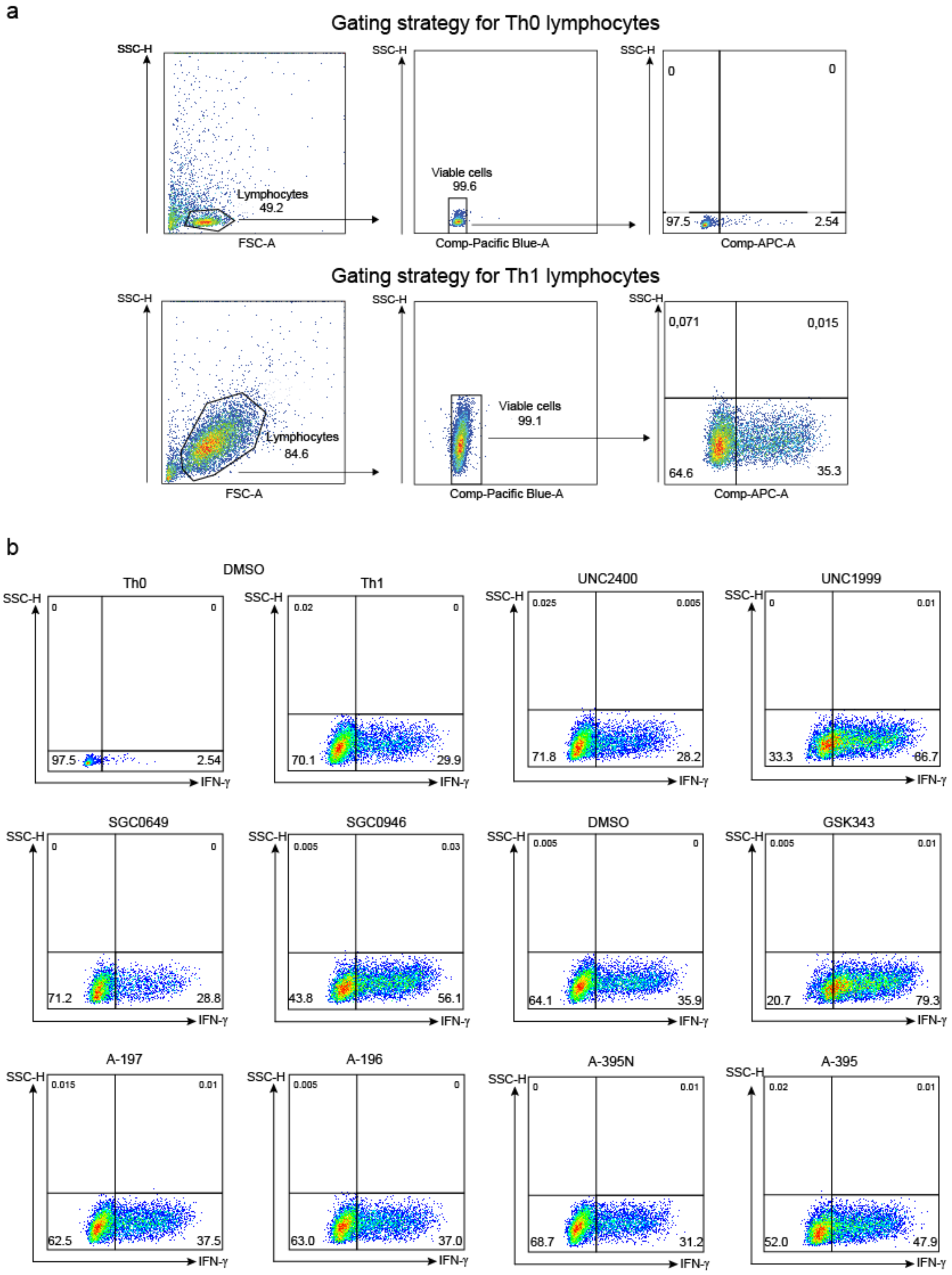

Supplementary Figure 4. FACS analysis of human Th1 polarized cells in the presence of indicated compounds or their corresponding controls. (a) Gating strategy to determine the frequency of IFN-ν producing Th0 and Th1 cells. (b) Naïve CD4+ T cells were isolated from PBMCs of 3 healthy donors and cultured for 4 days under Th0 or Th1 cell polarizing conditions in the presence of indicated compounds and their controls (1μM) and analyzed by FACS. Data shown is gated on viable CD4+ T cells. Related to Figs. 4c and 4d.

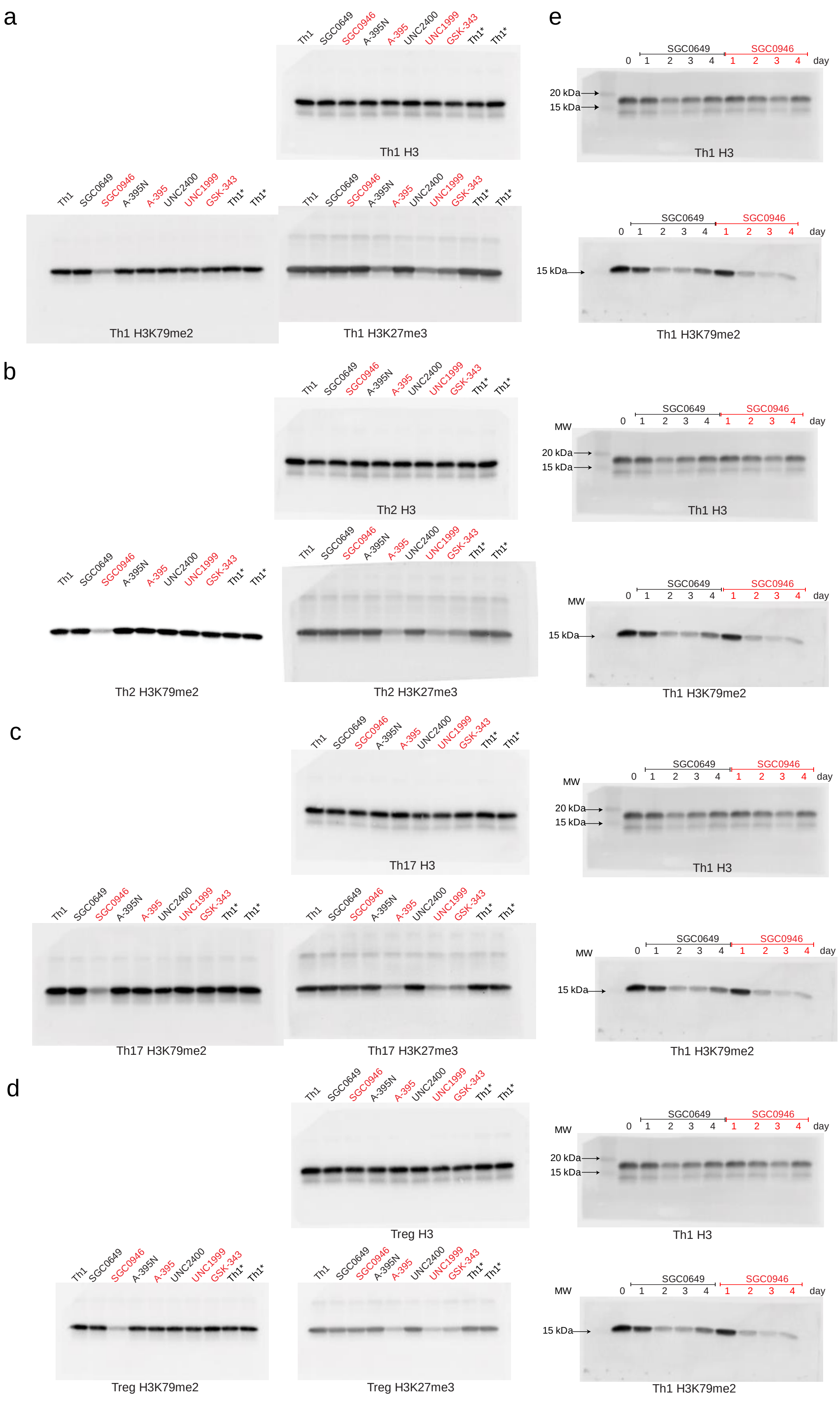

Supplementary Figure 5. Uncropped western blots illustrating the effect of indicated inhibitors (red) or control compounds (black) on the trimethylation of H3K27 and dimethylation of H3K79 in CD4+ T cells under Th1 cell-polarizing

conditions, the pan-H3 loading controls, and DOT1L-dependent regulation of H3K79me2. Western blots in Figs. 4e and 5a (in the main text) were generated using the same methods for histone extraction and western blotting including the batch of antibody. Since the gels and blots were very reproducible, when we generated the data shown in Fig. 4e (and Supplementary Figs 5 a-d), we had refrained from adding MW markers because we wanted to use the 2 rightmost lanes (labelled Th1*) for a study that is unrelated to this paper. Since the blots used for H3 in Fig. 5a (and Supplementary Fig. 5e) did have MW markers, the uncropped blots for both Figs. 4e and 5a (in the main text) are aligned according to H3 bands (to generate these composite images). Related to Figs. 4e and 5a.

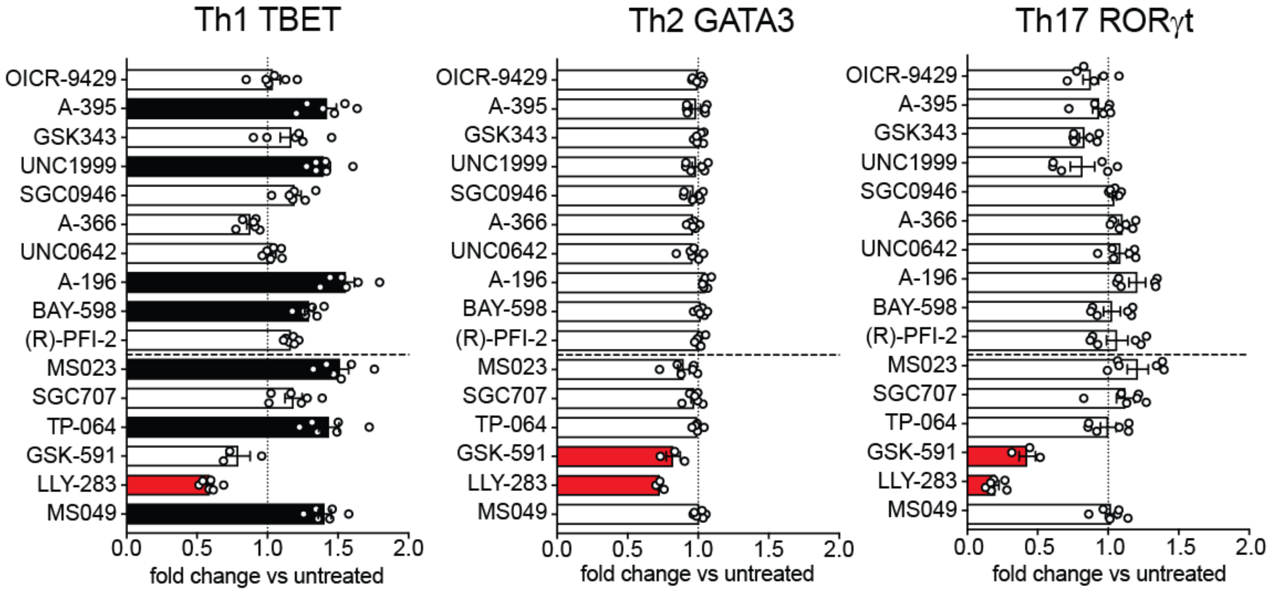

Supplementary Figure 6. Flow cytometric analysis of transcription factor expression in murine Th1 cells activated in the presence of indicated compounds or their corresponding controls. CD4^{+} T cells from the spleen and peripheral lymph nodes of mice were enriched and polarized under Th1, Th2 or Th17 cell-polarising conditions in the presence of indicated probes (1 μM) or their controls (where available) and analysed for expression of T-bet (Th1), Gata3 (Th2) or RORγt (Th17). Data shown is gated on viable CD4^{+} T cells and is the combined data from 2 independent experiments. Red bars indicate significant downregulation of the transcription factor, black bars indicate significant upregulation. Error bars represent SEM. Related to Fig. 6.

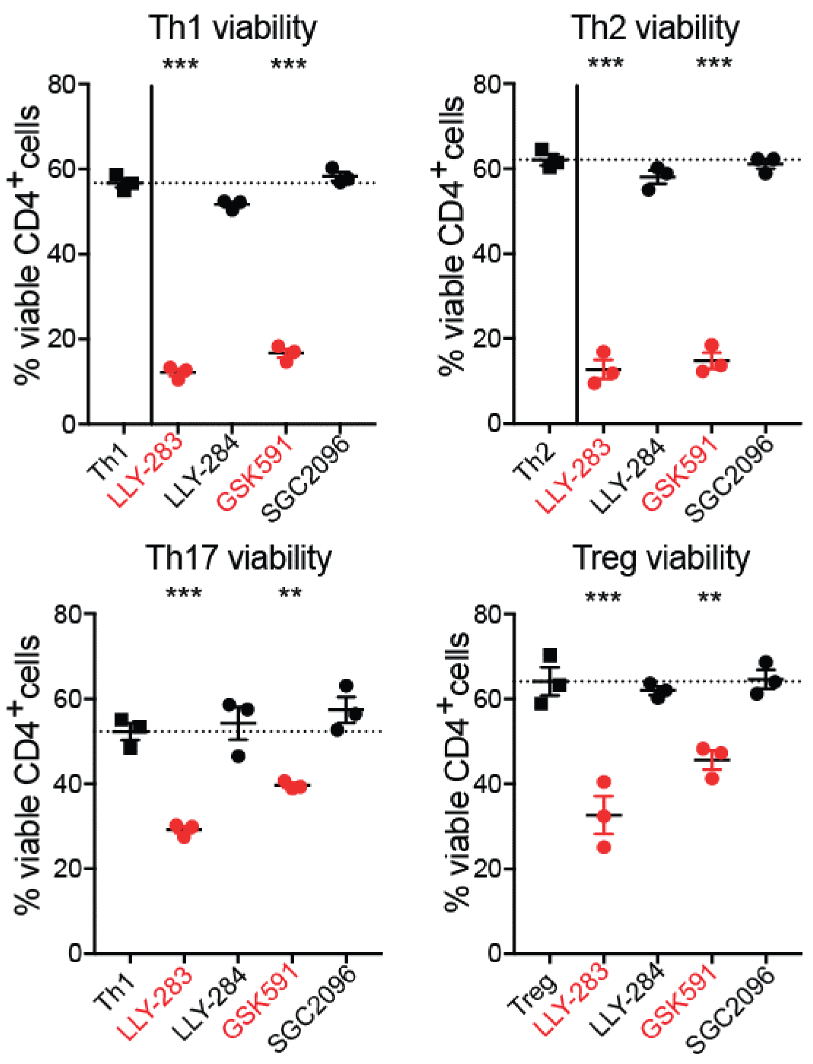

Supplementary Figure 7. Cell viability after 4 days under indicated polarizing conditions in the presence of PRMT5 inhibitors LLY-283 or GSK591 and their control compounds LLY-284 and SGC2096, respectively. Statistical significance between conditions was determined using one-way ANOVA (** $p \leq 0.01$, *** $p \leq 0.001$). Data is from 3 independent experiments. Error bars represent SEM. Related to Fig. 6.

SUPPLEMENTARY METHODS

CONTACT FOR REAGENT AND RESOURCE SHARING#

Domain: Protein target	Probe	Supplier‡	Control	Supplier	PubMed ID
MT*: DOT1L	SGC0946†	C,M,T	SGC0649	SGC	23250418
Kme binder: EED	A-395	M	A-395N	M	28135237
MT*: EZH2	GSK343	C,M,T	-	-	24900432
MT*: EZH2/H1	UNC1999	C,M,T	UNC2400	T	23614352
MT*: G9a/GLP	A-366	C,M,T	-	-	24900801
	UNC0642	C,M,T	-	-	24102134
MT*: PRMT type 1	MS023	C,M,T	MS094	SGC	26598975
MT*: PRMT3	SGC707	C,M,T	XY-1	C,T	25728001
MT*: PRMT4/6	MS049	C,M,T	MS049N	SGC	27584694
MT*: PRMT7	SGC3027	SGC	SGC3027N	SGC	
MT*: PRMT4	TP-064	M,T	TP-064N	SGC	29719619
	SKI-73¥	SGC	SKI-73N	SGC	
MT*: PRMT5	GSK591	C,M,T	SGC2096	SGC	26985292
	LLY-283	M	LLY-284	M	30034588
MT*: SETD7	(R)-PFI-2	C,M,T	(S)-PFI-2	C,M,T	25136132
MT*: SMYD2	BAY-598	C,M	BAY-369	SGC	27075367
MT*: SMYD3	BAY-6035	SGC	BAY-444	SGC	
MT*: SUV420H1/H2	A-196	C,M,T	A-197, SGC2043	SGC	28114273
Kme binder: WDR5	OICR-9429	C,M,T	OICR-0547	SGC	26167872

*MT - methyltransferase
¥Prodrug of SKI-72
‡Cayman Chemical (C), Millipore-Sigma (M), Tocris (T)
†Biotinylated probe (SGC2077) is available from the SGC (proberequests@thesgc.org).
#Further information about resources and reagents should be directed to Peter Brown (peterj.brown@utoronto.ca). SGC Probe collection is sold by Cayman Chemical (Cat # 17748).

Synthesis of reagents

Contents

Procedure for the Preparation of SGC2098 18
Procedure for the Preparation of SGC2043 19
Procedure for the Preparation of SGC2096 20
Procedure for the Preparation of SGC0649 20
Procedure for the preparation of SGC2077 21
Procedure for the Preparation of MTM7172 23
Procedure for the Preparation of UNC3815 26
Procedure for the Preparation of UNC3811 28
Procedure for the preparation of SGC1442 and SGC0784 29
Procedure for the preparation of (A-395)-biotin. 32
Procedure for the preparation of (A-395N)-biotin 34
Procedure for the preparation of SGC3185. 35

Chemistry General Procedures: Analytical thin-layer chromatography (TLC) was performed employing EMD Millipore 210-270 μm 60-F254 silica gel plates. The plates were visualized by exposure to UV light. Flash column chromatography was performed on a Teledyne ISCO Combi*Flash* Rf+ system equipped with a variable wavelength UV detector and a fraction collector using Redi*Sep* Rf normal or reverse phase silica columns. Nuclear Magnetic Resonance (NMR) spectra were acquired on a Bruker DRX-600 spectrometer or on a Varian Mercury spectrometer at 400 MHz. Chemical shifts are reported in parts per million (ppm, δ) scale relative to solvent residual peak (chloroform-d, ^{1}H: 7.26 ppm; methanol-d_4, ^{1}H: 3.31 ppm). ^{1}H NMR data are reported as follows: chemical shift, multiplicity (s = singlet, br s = broad singlet, d = doublet, t = triplet, q = quartet, p = pentet, m = multiplet, app = apparent), coupling constant, and integration. HPLC spectra for all compounds were acquired using an Agilent 6110 series system with a UV detector set to 254 nm. Samples were injected (5 μL) onto an Agilent Eclipse Plus, 4.6 Å~ 50 mm, 1.8 μm, C18 column at room temperature. Either with a linear gradient from 50% to 100% B (MeOH + 0.1% acetic acid) in 5.0 min was followed by pumping 100% B for another 2 min with A being H_2O + 0.1% acetic acid or by a linear gradient from 10% to 100% B (MeOH + 0.1% acetic acid) in 5.0 min was followed by pumping 100% B for another 2 min with A being H_2O + 0.1% acetic acid. Preparative HPLC was performed on an Agilent Prep 1200 series with a UV detector set to 254 nm. Samples were injected onto a Phenomenex Luna, 30x75 mm, 5 μM, C18 column at room temperature. The flow rate was 40 mL/min. A linear gradient was used with 10% (or 50%) MeOH (A) in 0.1% TFA in H_2O (B) to 100% MeOH (A). Mass spectrometry (MS) data were acquired in positive ion mode using an Agilent 6110 single-quadrupole mass spectrometer with an electrospray ionization (ESI) source. HRMS analysis was conducted on an Agilent Technologies G1969A high-resolution API-TOF mass spectrometer attached to an Agilent Technologies 1200 HPLC system. Samples were ionized by electrospray ionization (ESI) in positive mode. All biologically evaluated compounds had > 95% purity using the HPLC methods described above.

Procedure for the Preparation of SGC2098

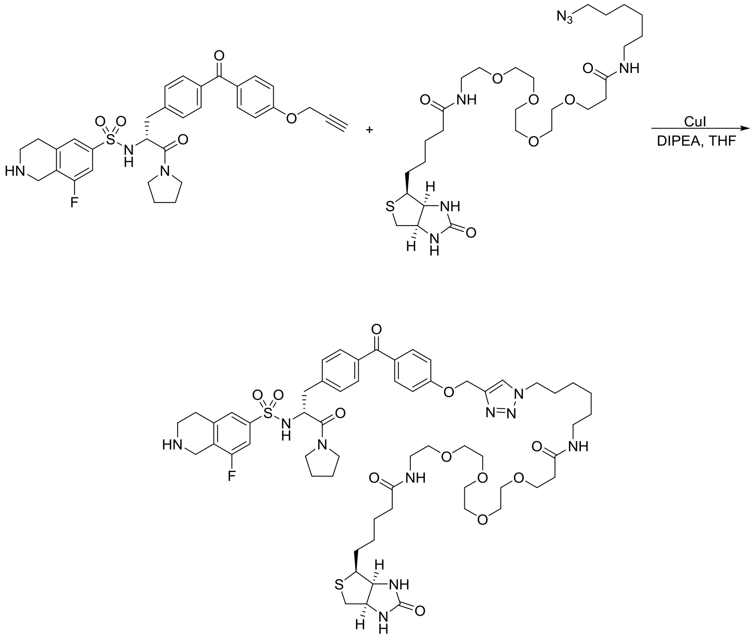

Procedure involves dissolving (*R*)-8-fluoro-*N*-(1-oxo-3-(4-(4-(prop-2-yn-1-yloxy)benzoyl)phenyl)-1-(pyrrolidin-1-yl)propan-2-yl)-1,2,3,4-tetrahydroisoquinoline-6-sulfonamide[1] (152 mg, 0.258 mmol) in THF (1.7 mL) at room temperature, followed by the addition of *N*-(6-azidohexyl)-1-(5-((3a*S*,4*S*,6a*R*)-2-oxohexahydro-1*H*-thieno[3,4-*d*]imidazol-4-yl)pentanamido)-3,6,9,12-tetraoxapentadecan-15-amide (170 mg, 0.271 mmol), DIPEA (220 □L, 1.29 mmol), and CuI (10 mg, 0.052 mmol). The resulting solution was stirred for 2 h prior to filtration of the entire mixture through a small plug of Celite® and subsequent removal of all solvents under reduced pressure. The residue was purified by column chromatography on silica gel using 0-100% MeOH/EtOAc as the eluent to yield **SGC2098** (90 mg, 29%). ^{1}H NMR (500 MHz, MeOD) δ 8.12 (s, 1H), 7.77 (d, *J* = 8.7 Hz, 2H), 7.64 – 7.60 (m, 2H), 7.34 (t, *J* = 6.5 Hz, 3H), 7.21 (d, *J* = 8.8 Hz, 1H), 7.16 (d, *J* = 8.8 Hz, 2H), 5.30 (s, 2H), 4.49 (dd, *J* = 12.7, 5.3 Hz, 2H), 4.43 (t, *J* = 7.0 Hz, 2H), 4.33 – 4.26 (m, 3H), 3.72 (dd, *J* = 10.9, 6.0 Hz, 4H), 3.54 (dd, *J* = 12.4, 5.6 Hz, 4H), 3.36 (dd, *J* = 11.3, 5.7 Hz, 4H), 3.20 – 3.13 (m, 6H), 2.70 (dd, *J* = 12.7, 5.4 Hz, 2H), 2.43 (dd, *J* = 11.1, 5.9 Hz, 4H), 2.21 (dd, *J* = 14.1, 7.1 Hz, 4H), 1.92 (d, *J* = 7.1 Hz, 4H), 1.73 – 1.55 (m, 12H), 1.52 – 1.29 (m, 18H); ^{13}C NMR (151 MHz, MeOD) δ 196.87, 176.11, 176.08, 173.85, 170.45, 166.04, 163.63, 144.33, 142.55, 141.39, 137.89, 133.57, 131.56, 130.91, 130.63, 125.56, 124.44, 124.01, 115.69, 111.78, 111.61, 96.86, 71.48, 71.45, 71.42, 71.38, 71.25, 71.21, 70.69, 68.36, 63.34, 62.60, 61.60, 57.00, 52.37, 51.35, 47.72, 47.00, 41.07, 40.31, 40.18, 37.62, 36.74, 31.18, 30.27, 30.16, 29.83, 29.77, 29.51, 27.49, 27.24, 27.08, 26.85, 24.97; LRMS (m/z): $[M+H]^+$ calcd. for $C_{59}H_{82}FN_{10}O_{12}S_2$, 1205.6; found, 1206.2.

Procedure for the Preparation of SGC2043

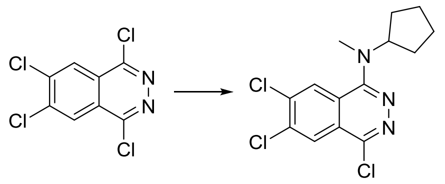

1,4,6,7-Tetrachlorophthalazine (1.35 g, 5.04 mmol) was dissolved in DMSO (13 mL) at room temperature, followed by the addition of *N*,*N*-diisopropylethylamine (1.14 mL, 6.55 mmol). The resulting mixture was then heated to 80 °C, followed by the dropwise addition of *N*-methylcyclopentanamine (0.500 g, 5.04 mmol) in DMSO (1 mL). After addition, the resulting solution was stirred for an additional 2 h prior to cooling to room temperature. The solution was stirred for 16 h at room temperature before dilution with brine (50 mL), and extraction with EtOAc (3 x 20 mL). The combined organic extracts were dried over anhydrous Na_2SO_4, filtered, and concentrated under reduced pressure. The residue of 4,6,7-trichloro-*N*-cyclopentyl-*N*-methylphthalazin-1-amine (1.62 g, 97%) was then dried under vacuum for 16 h, and used in the subsequent reaction without further purification; LRMS (m/z): $[M+H]^+$ calcd. for $C_{14}H_{15}Cl_3N_3$, 330.0; found, 331.6.

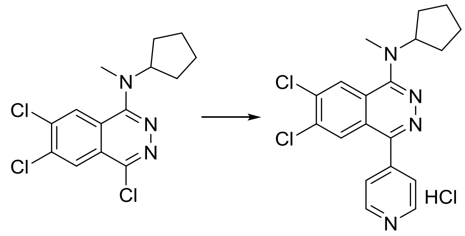

4,6,7-Trichloro-*N*-cyclopentyl-*N*-methylphthalazin-1-amine (400 mg, 1.21 mmol) was dissolved in dioxane/H_2O (4:1, 25 mL) at room temperature, followed by the addition of 4-pyridinylboronic acid (149 mg, 1.21 mmol), potassium carbonate (501 mg, 3.63 mmol), and solid-supported tetrakis(triphenylphosphine)palladium (0) (Aldrich # 511579, 0.5-0.9 mmol/g loading, 0.242 mg, 0.121 mmol). The resulting solution was then heated to 80 °C for 16 h prior to cooling to room temperature, dilution with brine (40 mL), and extraction with EtOAc (3 x 30 mL). The combined organic extracts were dried over anhydrous Na_2SO_4, filtered, and concentrated under reduced pressure. The residue was purified by flash column chromatography on silica gel (10-100% EtOAc/hexanes), and the appropriate product fraction collected prior to removal of all solvents under reduced pressure. The residue was then dissolved in aqueous HCl solution (1 N, 10 mL) before being washed with EtOAc (2 x 20 mL) to remove some triphenylphosphine contaminant. The aqueous layer was subsequently frozen and lyophilized over 16 h to yield 6,7-dichloro-*N*-cyclopentyl-*N*-methyl-4-(pyridin-4-yl)phthalazin-1-amine hydrochloride (**SGC2043**) (116 mg, 23%). ^{1}H NMR (500 MHz, DMSO-d_6) δ 9.01 (d, *J* = 5.7 Hz, 2H), 8.43 (s, 1H), 8.17 (d, *J* = 5.6 Hz, 2H), 8.15 (s, 1H), 4.66-4.59 (m, 1H), 3.22 (s, 3H), 2.09-2.00 (m, 2H), 1.88-1.73 (m, 4H), 1.67-1.56 (m, 2H); ^{13}C NMR (126 MHz, DMSO) δ 157.70 (s), 148.62 (s), 144.40 (s), 136.91 (s), 135.14 (s), 128.52 (s), 128.11 – 127.62 (m), 127.62 – 126.95 (m), 126.92 (s), 120.63 (s), 63.10 (s), 35.90 (s), 28.99 (s), 24.64 (s); LRMS (m/z): $[M+H]^+$ calcd. For $C_{19}H_{19}Cl_2N_4$, 373.1; found, 374.2.

Procedure for the Preparation of SGC2096

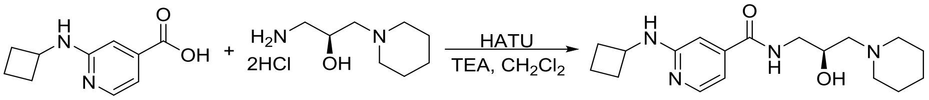

Procedure involves suspending 2-(cyclobutylamino)isonicotinic acid (270 mg, 1.40 mmol) in CH_2Cl_2 (6 mL) at room temperature, followed by the addition of (*S*)-1-amino-3-(piperidin-1-yl)propan-2-ol dihydrochloride (390 mg, 1.69 mmol), HATU (532 mg, 1.40 mmol), and DMF (2 mL). Triethylamine (0.59 mL, 4.20 mmol) was then added, and the resulting solution stirred for 16 h prior to dilution with brine (15 mL), adjustment to pH = 13 with aqueous sodium hydroxide solution, and extraction of the aqueous layer with EtOAc (3 x 20 mL). The combined organic extracts were dried over anhydrous Na_2SO_4, filtered, and concentrated under reduced pressure. The residue was purified by column chromatography on silica gel using 0-40% MeOH/EtOAc as the eluent to yield (*S*)-2-(cyclobutylamino)-*N*-(2-hydroxy-3-(piperidin-1-yl)propyl)isonicotinamide (235 mg, 51%). ^{1}H NMR (500 MHz, DMSO-d_6) δ 8.43 (br t, 1H), 8.03 (d, J = 5.0 Hz, 1H), 6.96 (d, J = 7.5 Hz, 1H), 6.81 (d, J = 5.0 Hz, 1H), 6.77 (s, 1H), 4.34-4.21 (m, 1H), 3.85-3.75 (m, 1H), 3.38-3.30 (m, 2H), 3.21-3.12 (m, 1H), 2.48-2.35 (br m, 4H), 2.34-2.22 (br m, 4H), 1.93-1.82 (m, 2H), 1.75-1.64 (m, 2H), 1.55-1.44 (br m, 4H), 1.41-1.32 (br s, 2H); ^{13}C NMR (126 MHz, DMSO) δ 157.70 (s), 148.62 (s), 144.40 (s), 136.91 (s), 135.14 (s), 128.52 (s), 128.11 – 127.62 (m), 127.62 – 126.95 (m), 126.92 (s), 120.63 (s), 63.10 (s), 35.90 (s), 28.99 (s), 24.64 (s); LRMS (m/z): $[M+H]^+$ calcd. for $C_{18}H_{29}N_4O_2$, 333.2; found, 333.2.

Procedure for the Preparation of SGC0649

The procedure described for the synthesis of **SGC0946** (Supplementary Information, *Nature Communications*, DOI: 10.1038/ncomms2304) was also used for the synthesis of **SGC0649**. The only difference between the two procedures involved replacement of 1-(4-(*tert*-butyl)phenyl)-3-(3-hydroxypropyl)urea with 4-(*tert*-butyl)-*N*-(3-hydroxypropyl)benzamide, the synthesis of which is described below.

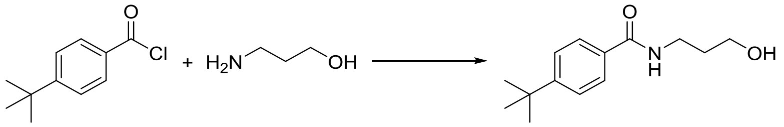

To a solution of 3-aminopropan-1-ol (7.78 mL, 101.7 mmol) and triethylamine (3.54 mL, 25.4 mmol) in CH_2Cl_2 (50 mL) at 0 °C was added 4-(*tert*-butyl)benzoyl chloride (4.97 mL, 25.4 mmol) dropwise over 10 minutes. The resulting solution was warmed to room temperature, and stirred for an additional 16 hours before being diluted with H_2O (50 mL). After the layers were separated, the organic layer was washed with HCl solution (1 M, 2 x 25 mL), dried with anhydrous Na_2SO_4, filtered, and concentrated under reduced pressure to afford the title compound as a colorless oil (4.61 g, 77%). The purity of this material was assessed as >95% by ^{1}H NMR, and used in the subsequent reaction without further purification. ^{1}H NMR (500 MHz, $CDCl_3$) δ 7.74 (d, J = 8.4 Hz,

2H), 7.43 (d, J = 8.5 Hz, 2H), 7.06 (s, 1H), 3.74 – 3.68 (m, 2H), 3.64 – 3.59 (m, 2H), 1.78 (dt, J = 11.6, 5.8 Hz, 2H), 1.33 (s, 9H); LRMS (m/z): $[M+H]^+$ calcd. for $C_{14}H_{22}NO_2$, 236.2; found, 236.2.

***N*-(3-((((2*R*,3*S*,4*R*,5*R*)-5-(4-Amino-5-bromo-7*H*-pyrrolo[2,3-*d*]pyrimidin-7-yl)-3,4-dihydroxytetrahydrofuran-2-yl)methyl)(isopropyl)amino)propyl)-4-(*tert*-butyl)benzamide (SGC0649)**

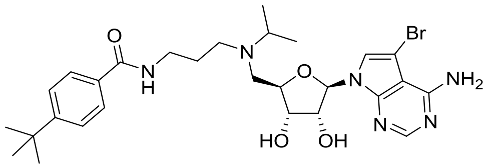

SGC0649

The title compound was isolated as a white powder. ^1H NMR (500 MHz, DMSO-d_6) δ 8.43 (br s, 1H), 8.11 (s, 1H), 7.74 (d, J = 8.3 Hz, 2H), 7.69 (s, 1H), 7.41 (d, J = 8.3 Hz, 2H), 6.81 (br s, 1H), 6.09 (d, J = 5.5 Hz, 1H), 5.48 (br s, 1H), 4.42 (br s, 1H), 4.20 – 3.91 (m, 2H), 3.34 – 3.21 (m, 2H), 3.17 (s, 1H), 2.64 (br s, 2H), 1.92 (s, 2H), 1.72 (br s, 2H), 1.28 (s, 9H), 1.04 (s, 3H), 0.98 (s, 3H); ^{13}C NMR (126 MHz, DMSO-d_6) δ 172.46, 158.60 (q, J = 30.8 Hz), 157.42, 154.22, 153.03, 150.27, 132.32, 127.41 (2), 125.38 (2), 122.29, 117.78 (q, J = 300.2 Hz). 101.53, 87.61, 73.42, 72.13, 52.75, 49.06, 48.81, 37.95, 35.00, 31.41 (3), 21.54, 17.24 (2); LRMS (m/z): $[M+H]^+$ calcd. for $C_{28}H_{40}BrN_6O_4$, 603.2; found, 603.8.

Procedure for the preparation of *SGC2077*

The procedure described for the synthesis of 1-(1-amino-18-(((2*R*,3*S*,4*R*,5*R*)-5-(4-amino-5-bromo-7*H*-pyrrolo[2,3-*d*]pyrimidin-7-yl)-3,4-dihydroxytetrahydrofuran-2-yl)methyl)-3,6,9,12,15-pentaoxa-18-azahenicosan-21-yl)-3-(4-(*tert*-butyl)phenyl)urea (**12**) (Supplementary Information, *Nature Structural & Molecular Biology*, DOI: 10.1038/nsmb.3249) was also used for the synthesis of **SGC2077**. The amine (**12**) was prepared, and then biotinylated according to the procedure described below.

***N*-(6-(((2*R*,3*S*,4*R*,5*R*)-5-(4-Amino-5-bromo-7*H*-pyrrolo[2,3-*d*]pyrimidin-7-yl)-3,4-dihydroxytetrahydrofuran-2-yl)methyl)-1-((4-(*tert*-butyl)phenyl)amino)-1-oxo-9,12,15,18,21-pentaoxa-2,6-diazatricosan-23-yl)-5-((3a*S*,4*S*,6a*R*)-2-oxohexahydro-1H-thieno[3,4-*d*]imidazol-4-yl)pentanamide (SGC2077)**

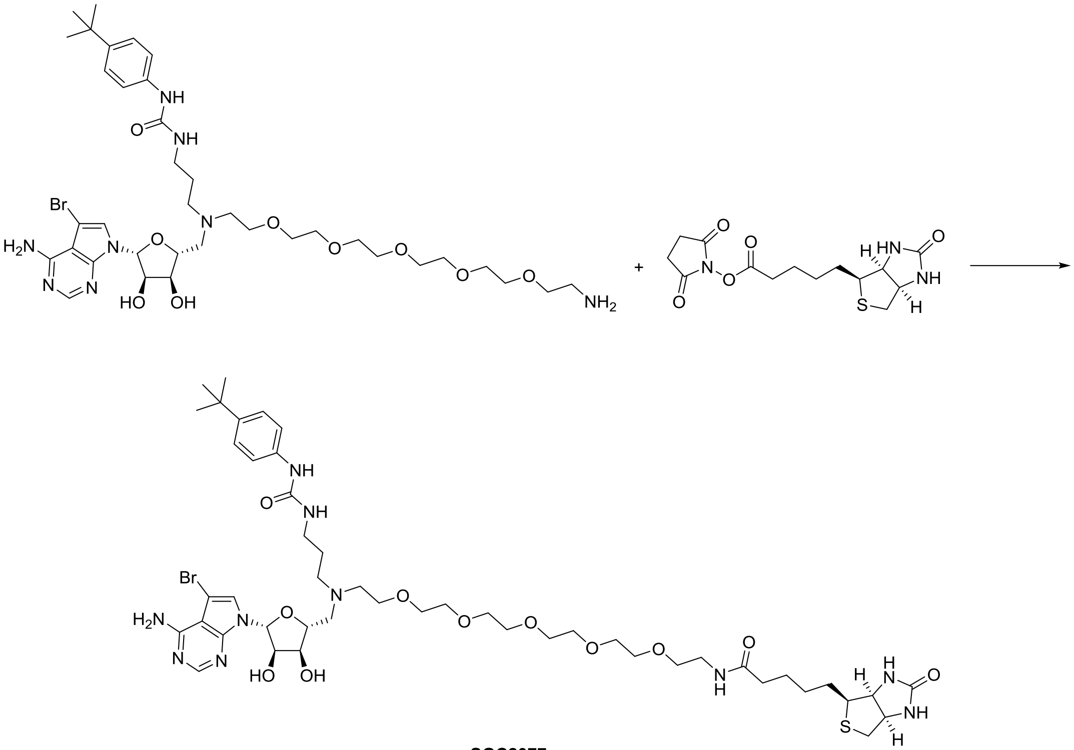

SGC2077

To a solution of 1-(1-amino-18-(((2*R*,3*S*,4*R*,5*R*)-5-(4-amino-5-bromo-7*H*-pyrrolo[2,3-*d*]pyrimidin-7-yl)-3,4-dihydroxytetrahydrofuran-2-yl)methyl)-3,6,9,12,15-pentaoxa-18-azahenicosan-21-yl)-3-(4-(*tert*-butyl)phenyl)urea (0.404 g, 0.481 mmol) in DMF (5.7 mL) at room temperature was added triethylamine (1 mL) until pH = 9. Biotin-OSu (0.197 g, 0.577 mmol) was then added, and the resulting solution stirred for 2 hours. The reaction mixture was diluted with saturated aqueous $NaHCO_3$ solution (25 mL), and extracted with CH_2Cl_2 (3 x 15 mL). The combined organic extracts were dried with anhydrous Na_2SO_4, filtered, and concentrated under reduced pressure. The residue was purified by column chromatography on silica gel (Biotage SNAP 25 g column, 5-40% MeOH/EtOAc as the eluent, 26 CV) to afford the title compound as a white powder (0.220 g, 43%). ^{1}H NMR (500 MHz, DMSO-d_6) δ 8.45 (s, 1H), 8.11 (s, 1H), 7.83 (s, 1H), 7.66 (s, 1H), 7.29 (d, *J* = 8.5 Hz, 2H), 7.21 (d, *J* = 8.4 Hz, 2H), 6.79 (br s, 2H), 6.42 (s, 1H), 6.36 (s, 1H), 6.25 (s, 1H), 6.06 (d, *J* = 5.5 Hz, 1H), 5.42 (br s, 1H), 5.29 (br s, 1H), 4.39 – 4.33 (m, 1H), 4.33 – 4.27 (m, 1H), 4.15 – 4.10 (m, 1H), 4.04 – 4.00 (m, 1H), 3.94 – 3.88 (m, 1H), 3.51 – 3.45 (m, 19H), 3.39 (t, *J* = 5.8 Hz, 3H), 3.23 – 3.15 (m, 2H), 3.13 – 3.04 (m, 3H), 2.80 (td, *J* = 12.4, 5.1 Hz, 2H), 2.67 – 2.60 (m, 3H), 2.58 (d, *J* = 12.5 Hz, 1H), 2.06 (t, *J* = 7.3 Hz, 2H), 1.66 – 1.41 (m, 6H), 1.34 – 1.26 (m, 2H), 1.25 (s, 9H); ^{13}C NMR (126 MHz, MeOD) δ 176.08, 166.06, 158.61, 158.42, 153.45, 150.92, 146.31, 138.25, 126.52, 123.32, 120.21, 103.18, 89.58, 89.19, 83.53, 75.30, 73.41, 71.52, 71.48, 71.45, 71.30, 71.22, 70.60, 63.35, 61.60, 56.98, 55.20, 41.05, 40.45, 40.36, 39.55, 36.74, 35.01, 31.90, 29.76, 29.50, 26.83; LRMS (m/z): $[M+H]^+$ calcd. for $C_{47}H_{74}BrN_{10}O_{11}S$, 1065.4; found, 1067.8.

Procedure for the Preparation of MTM7172

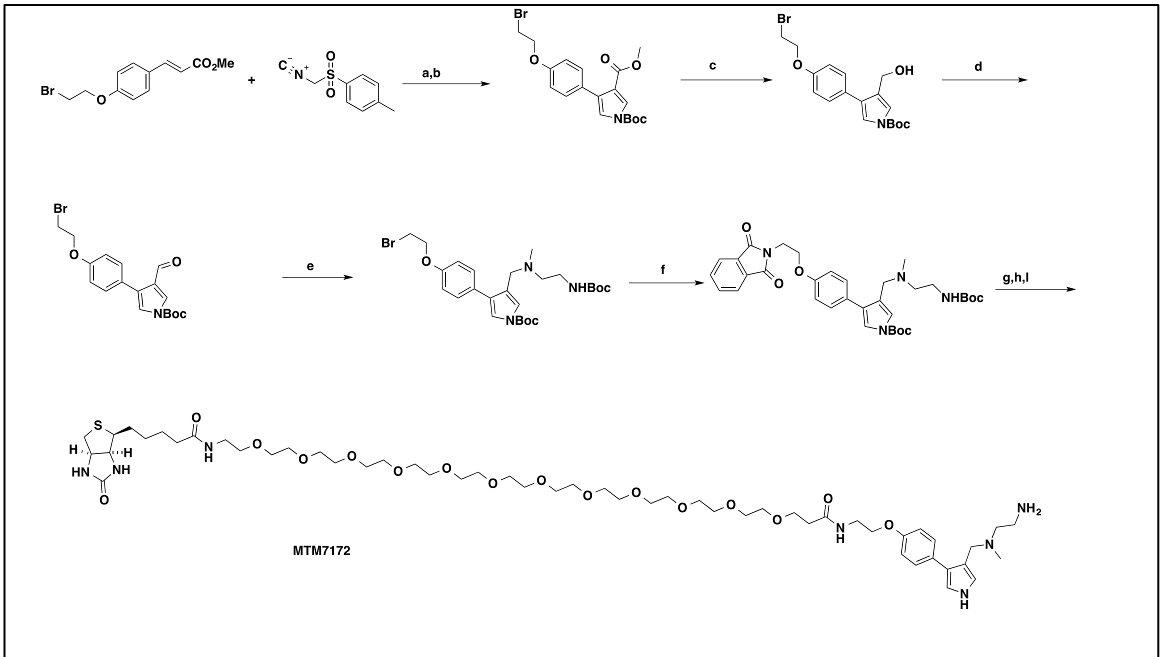

Reagents and conditions: (a) NaH, DMF/THF; (b) Boc_2O, Et_3N, DMAP, DCM, 15% over two steps; (c) DIBALH, THF, -78°C, 83%; (d) DMP, DCM, 93%; (e) *tert*-butyl (2-(methylamino)ethyl)carbamate, $NaBH(OAc)_3$, DCM, 91%; (f) potassium 1,3-dioxoisoindolin-2-ide, DMF, 100°C, 73%; (g) Hydrazine, EtOH, reflux; (h) *tert-butyl 3-(((2-((tert-butoxycarbonyl)amino)ethyl)(methyl)amino)methyl)-4-(4-(2-(1,3-dioxoisoindolin-2-yl)ethoxy)phenyl)-1H-pyrrole-1-carboxylate*, EDCI, HOAt, NMM, DMSO; (l) HCl/Methanol, 8% over three steps.

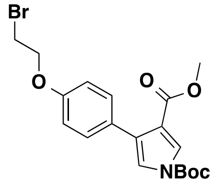

Synthesis of 1-(*tert*-butyl) 3-methyl 4-(4-(2-bromoethoxy)phenyl)-1*H*-pyrrole-1,3-dicarboxylate: To a mixture of sodium hydride (380 mg, 9.47 mmol, 60% in mineral oil) in 8 mL of tetrahydrofuran (THF) and 8 mL of N, N-dimethylformaldehyde (DMF) cooled by ice bath, was added dropwise during 15 min the solution of methyl (*E*)-3-(4-(2-bromoethoxy)phenyl)acrylate (900 mg, 3.15 mmol), 1-((isocyanomethyl)sulfonyl)-4-methylbenzene (616 mg, 3.15 mmol) in 8 mL of THF and 8 mL of DMF. The mixture was stirred for additional 1 h before carefully adding water to quench the reaction. The solution was extracted with ethyl acetate (EtOAc) (3 x 20 mL), and combined organic phase was dried over anhydrous sodium sulfate and concentrated under reduced pressure. The residue was used for next step without purification. The residue was dissolved in 50 mL of dichloromethane, and triethylamine (0.4 mL, 3.15 mmol), and 4-(dimethylamino)pyridine (38 mg, 0.31 mmol) were added. To the cooled solution in ice bath, was added di-*tert*-butyl dicarbonate (686 mg, 3.15 mmol) in portions. The solution was stirred overnight and quenched by water. The mixture was extracted with dichloromethane (3 x 20 mL), combined organic phase was dried over anhydrous sodium sulfate and concentrated under reduced pressure. The residue was purified by flash chromatography on silica gel column with eluent (EtOAc/hexane, 0-30%) to give an oil (200 mg, 15%). ^1H NMR (600 MHz, chloroform-d) δ 7.88 (s,

1H), 7.40 (d, *J* = 8.7 Hz, 2H), 7.19 (s, 1H), 6.91 (d, *J* = 8.7 Hz, 2H), 4.31 (t, *J* = 6.3 Hz, 2H), 3.76 (s, 3H), 3.65 (t, *J* = 6.3, 3.2 Hz, 2H), 1.62 (s, 9H).

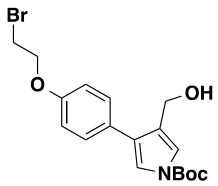

Synthesis of *tert*-butyl 3-(4-(2-bromoethoxy)phenyl)-4-(hydroxymethyl)-1*H*-pyrrole-1-carboxylate: To a solution of 1-(*tert*-butyl) 3-methyl 4-(4-(2-bromoethoxy)phenyl)-1*H*-pyrrole-1,3-dicarboxylate (200 mg, 0.47 mmol) in 10 mL of tetrahydrofuran cooled at -78°C, was added dropwise diisobutylaluminium hydride (DIBAL-H)(3 mL, 1 M in hexane). After completion of the reaction monitored by TLC, 1 mL of methanol was added cautiously to quench the reaction. Then 10 mL of saturated aqueous sodium potassium tartrate was added and the resulting mixture was stirred until transparent phase observed. The solution extracted with ethyl acetate and dried over anhydrous sodium sulfate, concentrated under reduced pressure. The residue was purified by flash chromatography on silica gel column with eluent (EtOAc/hexane, 0-50%) to give an oil (150 mg, 83%). ^{1}H NMR (600 MHz, chloroform-d) δ 7.47 (d, *J* = 8.7 Hz, 2H), 7.34 – 7.21 (m, 2H), 6.94 (d, *J* = 8.8 Hz, 2H), 4.60 (s, 2H), 4.31 (t, *J* = 6.3 Hz, 2H), 3.65 (t, *J* = 6.3 Hz, 2H), 1.60 (s, 9H).

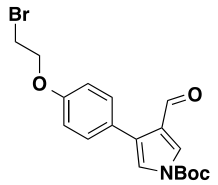

Synthesis of *tert*-butyl 3-(4-(2-bromoethoxy)phenyl)-4-formyl-1*H*-pyrrole-1-carboxylate: To a solution of *tert*-butyl 3-(4-(2-bromoethoxy)phenyl)-4-(hydroxymethyl)-1*H*-pyrrole-1-carboxylate (200 mg, 0.5 mmol) in 10 mL of dichloromethane, was added Dess-Martin periodinane (318 mg, 0.75 mmol) in portion. After completion of the reaction as TLC showed, the mixture was purified by flash chromatography on silica gel column with eluent (EtOAc/hexane, 0-40%) to give an oil (140 mg, 93%). ^{1}H NMR (600 MHz, chloroform-d) δ 9.91 (s, 1H), 7.91 (s, 1H), 7.44 (d, 2H), 7.27 (s, 1H), 6.95 (d, *J* = 7.9 Hz, 2H), 4.33 (t, *J* = 6.3 Hz, 2H), 3.66 (t, *J* = 6.3 Hz, 2H), 1.64 (s, 9H).

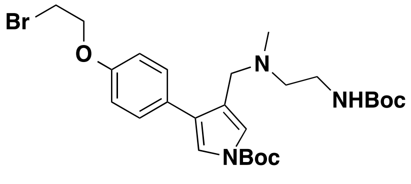

Synthesis of *tert*-butyl 3-(4-(2-bromoethoxy)phenyl)-4-(((2-((*tert*-butoxycarbonyl)amino)ethyl)(methyl)amino)methyl)-1*H*-pyrrole-1-carboxylate: To a solution of *tert*-butyl 3-(4-(2-bromoethoxy)phenyl)-4-formyl-1*H*-pyrrole-1-carboxylate (140 mg, 0.36 mmol) and *tert*-butyl (2-(methylamino)ethyl)carbamate (72.8 mg, 0.42 mmol) in 10 mL of dichloromethane, was added sodium triacetoxyborohydride (127 mg, 0.6 mmol). The mixture was stirred overnight. Saturated aqueous sodium bicarbonate was added and extracted with dichloromethane. The combined organic phase was dried over

sodium sulfate and concentrated under reduced pressure. The residue was purified by flash chromatography on silica gel column with eluent (EtOAc/hexane, 0-50%) to give an oil (180 mg, 91%). ^{1}H NMR (600 MHz, chloroform-d) δ 7.48 (d, *J* = 8.6 Hz, 2H), 7.24 (s, 1H), 7.15 (s, 1H), 6.94 (d, *J* = 8.3 Hz, 2H), 4.78 (s, 1H), 4.31 (t, *J* = 6.4 Hz, 2H), 3.64 (t, *J* = 6.3 Hz, 2H), 3.36 (s, 2H), 3.18 – 3.12 (m, 2H), 2.45 (t, *J* = 6.0 Hz, 2H), 2.16 (s, 3H), 1.60 (s, 9H), 1.43 (s, 9H).

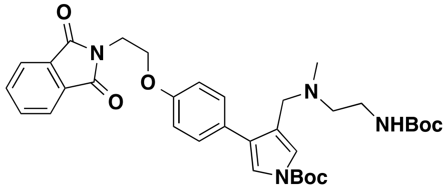

Synthesis of *tert*-butyl 3-(((2-((*tert*-butoxycarbonyl)amino)ethyl)(methyl)amino)methyl)-4-(4-(2-(1,3-dioxoisoindolin-2-yl)ethoxy)phenyl)-1*H*-pyrrole-1-carboxylate: *tert*-butyl 3-(4-(2-bromoethoxy)phenyl)-4-(((2-((*tert*-butoxycarbonyl)amino)ethyl)(methyl)amino)methyl)-1*H*-pyrrole-1-carboxylate (180 mg, 0.33 mmol) and potassium 1,3-dioxoisoindolin-2-ide (57.4 mg, 0.31 mmol) were dissolved into N,N-dimethylformaldehyde (DMF). The solution was heated to 100 °C and stirred overnight before the addition of water. The solution was extracted with ethyl acetate and combined organic phase was dried over anhydrous sodium sulfate, concentrated under reduced pressure. The residue was purified by flash chromatography on silica gel with eluent (EtOAc/hexane, 0-50%) to give a solid (140 mg, 73%). ^{1}H NMR (600 MHz, chloroform-d) δ 7.85 (dd, *J* = 5.4, 3.0 Hz, 2H), 7.71 (dd, *J* = 5.5, 3.0 Hz, 2H), 7.42 (d, *J* = 8.3 Hz, 2H), 7.19 (s, 1H), 7.13 (s, 1H), 6.89 (d, *J* = 8.8 Hz, 2H), 4.77 (s, 1H), 4.24 (t, *J* = 5.8 Hz, 2H), 4.11 (t, *J* = 6.6, 4.8 Hz, 2H), 3.33 (s, 2H), 3.17 – 3.09 (m, 2H), 2.42 (t, *J* = 5.9 Hz, 2H), 2.14 (s, 3H), 1.59 (s, 9H), 1.39 (s, 9H).

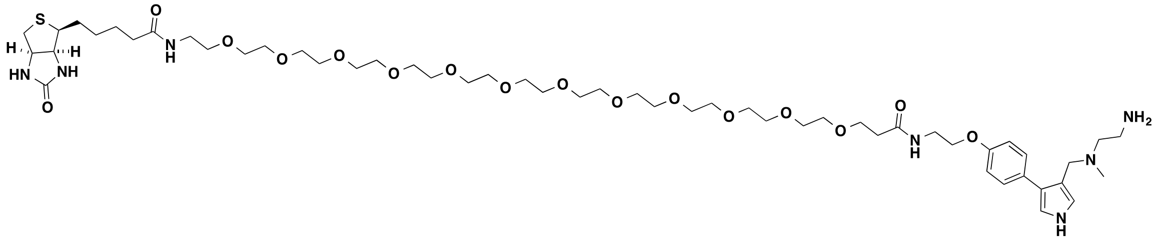

Synthesis of MTM7172: To a solution of *tert*-butyl 3-(((2-((*tert*-butoxycarbonyl)amino)ethyl)(methyl)amino)methyl)-4-(4-(2-(1,3-dioxoisoindolin-2-yl)ethoxy)phenyl)-1*H*-pyrrole-1-carboxylate (140 mg, 0.23 mmol) in 5 mL of ethanol, was added aqueous hydrazine (45.7 mg, 0.5 mmol, 35 wt. %), and the mixture was heated to reflux for 2 h. The volatile was removed under reduced pressure. The residue was used for next step without purification. To a solution of the crude residue, Biotin-PEG12-acid (84 mg, 0.1 mmol), (1-ethyl-3-(3-dimethylaminopropyl)carbodiimide) (29 mg, 0.15 mmol), and 1-hydroxy-7-azabenzo-triazole (20 mg, 0.15 mmol) in 5 mL of dimethylsulfoxide, was added 4-methylmorpholine (30 mg, 0.3 mmol). The solution was stirred overnight and purified by preparative HPLC (10%-100% methanol / 0.1% TFA in H_2O) to give oil. The oil was treated with 2 mL of dicloromethane and 2 mL of trifluoroacetic acid for 3 hours before the volatile was removed. The residue was lyophilized into oil as a TFA salt (20 mg, 8%). ^{1}H NMR (600 MHz, D_2O) δ 10.43 (s, 1H), 7.20 (d, *J* = 8.2 Hz, 2H), 7.00 (s, 1H), 6.91 (d, *J* = 8.1 Hz, 2H), 6.82 (s, 1H),

4.40 (dd, J = 8.0, 4.9 Hz, 1H), 4.31 (q, J = 13.6 Hz, 2H), 4.21 (dd, J = 8.1, 4.5 Hz, 1H), 3.99 (t, J = 5.2 Hz, 2H), 3.75 – 3.28 (m, 51H), 3.19 (t, J = 5.4 Hz, 3H), 3.11 (dt, J = 9.7, 4.9 Hz, 1H), 3.06 – 2.88 (m, 3H), 2.78 (dd, J = 13.1, 5.0 Hz, 1H), 2.58 (d, J = 13.1 Hz, 1H), 2.47 (s, 3H), 2.36 (t, J = 6.0 Hz, 2H), 2.07 (t, J = 7.3 Hz, 2H), 1.60 – 1.31 (m, 3H), 1.28 – 1.13 (m, 2H). ^{13}C NMR (201 MHz, D_2O) δ 176.7, 174.3, 165.2, 157.1, 129.9 (2C), 127.9, 124.3, 122.4, 117.7, 115.5 (2C), 107.2, 69.6 (21C), 69.4, 68.8, 66.7, 66.7, 62.1, 60.2, 55.4, 51.9, 50.2, 39.7, 39.1, 38.9, 38.8, 36.1 35.4, 33.8, 27.9, 27.7, 25.1; HRMS (m/z): $[M+H]^+$ calcd. for $C_{53}H_{92}N_7O_{16}S$, 1114.6316; found, 1114.6276.

Procedure for the Preparation of UNC3815

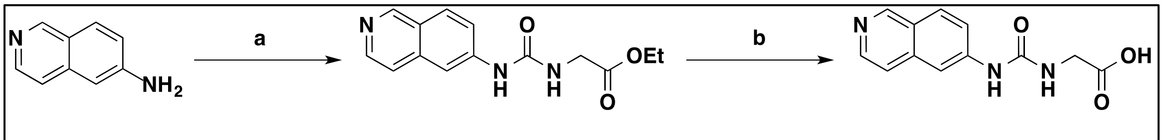

Reagents and conditions: (a) Ethyl isocyanatoacetate, DMF/DCM, RT; (b) NaOH, H_2O/MeOH, RT, 57% (2 steps)

Synthesis of (isoquinolin-6-ylcarbamoyl)glycine: To a stirring solution of 6-aminoisoquinoline (1.2 g, 8.32 mmol) in mixture of dichloromethane (DCM) and DMF (30 mL & 10 mL) was added ethyl isocyanatoacetate (2.80 mL, 25 mmol) and the resulting mixture was stirred 18 hours at room temperature. After removal of volatiles, crude mixture was purified by flash column chromatography (gradient from 100% dichloromethane to 10% methanol in dichloromethane) to yield desired ethyl ester as pale yellow solid which was re-suspended in Methanol (48 mL) and water (16 mL) followed by addition of 1N solution of NaOH (24 mL). The resulting clear mixture was then stirred at room temperature 16 hours. After concentration of the mixture under reduced pressure crude mixture was purified by reverse phase flash column chromatography (gradient from 100% water with 0.1 %TFA to 10% methanol) to yield desired (isoquinolin-6-ylcarbamoyl)glycine as a TFA salt (2.04 g, 57% over 2 steps). ^{1}H NMR (400 MHz, Methanol-d_4) δ 9.40 (s, 1H), 8.46 (d, J = 2.1 Hz, 1H), 8.36-8.30 (m, 2H), 8.15 (d, J = 6.7 Hz, 1H), 7.86 (dd, J = 9.0, 2.1 Hz, 1H), 4.01 (s, 2H); LRMS (m/z): $[M+H]^+$ calcd. for $C_{12}H_{12}N_3O_3$, 246.1; found, 246.1.

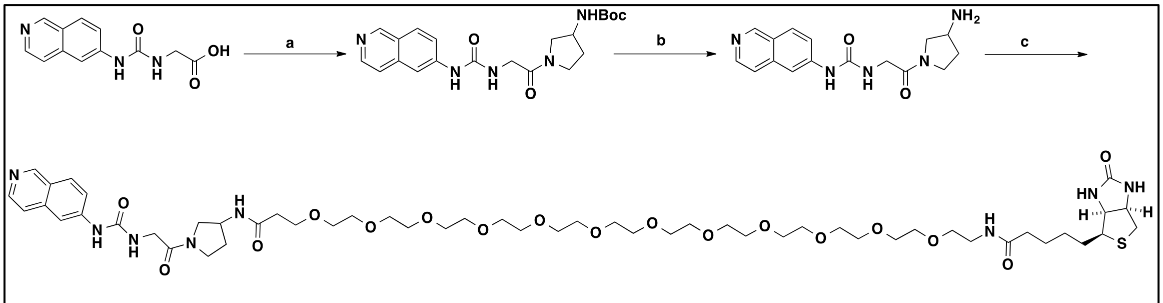

Reagents and conditions: (a) 3-Boc-aminopyrrolidine, EDC.HCl, HOAt, NMM, DMF, RT, 86%; (b) TFA, DCM, 0°C, 54% ; (c) Biotin-PEG-12-acid, HATU, DIPEA, DMF, RT, 87%

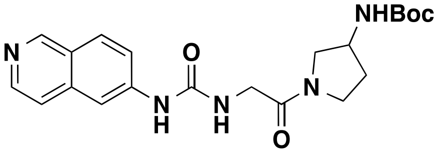

Synthesis of *tert*-butyl (1-((isoquinolin-6-ylcarbamoyl)glycyl)pyrrolidin-3-yl)carbamate: To a stirring mixture of (isoquinolin-6-ylcarbamoyl)glycine (60 mg, 0.17 mmol) in DMF (1.5 mL) was added N-(3-Dimethylaminopropyl)-N′-ethylcarbodiimide hydrochloride (EDC.HCl) (64.0 mg, 0.33 mmol); 1-Hydroxy-7-azabenzotriazole (HOAt) (35 mg, 0.25 mmol) and 3-Boc-aminopyrrolidine (62.2 mg, 0.33 mmol) followed by N-Methylmorpholine (NMM) (38 μL, 0.34 mmol) and resulting mixture was stirred 18 hours at room temperature. The volatiles were removed under reduced pressure and the crude reaction mixture was purified by flash column chromatography to give white solid (52.7 mg, 76%). ^{1}H NMR (400 MHz, Methanol-d_4) δ 9.01 (d, *J* = 0.9 Hz, 1H), 8.27 (d, *J* = 5.9 Hz, 1H), 8.06 (d, *J* = 2.0 Hz, 1H), 7.94 (d, *J* = 8.9 Hz, 1H), 7.60 (d, *J* = 5.9 Hz, 1H), 7.56 (dd, *J* = 8.9, 2.1 Hz, 1H), 4.24-4.10 (m, 1H), 4.10-3.96 (m, 2H), 3.78-3.49 (m, 3H), 3.41-3.34 (m, 1H), 2.27-2.07 (m, 1H), 2.02-1.84 (m, 1H), 1.45 (br s, 9H); LRMS (m/z): $[M+H]^+$ calcd. for $C_{21}H_{28}N_5O_4$, 414.2; found, 414.2.

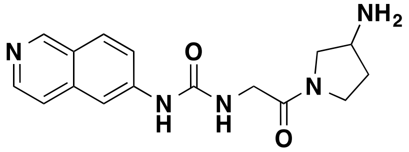

Synthesis of 1-(2-(3-aminopyrrolidin-1-yl)-2-oxoethyl)-3-(isoquinolin-6-yl)urea: To the stirring mixture of *tert*-butyl (1-((isoquinolin-6-ylcarbamoyl)glycyl)pyrrolidin-3-yl)carbamate (52.7 mg, 0.127 mmol) in dicholoromethane (1 mL) was added trifluoroacetic acid (TFA) (0.25 mL) at 0 °C and resulting mixture was stirred at 0 °C for 3.5 hrs. The volatiles were removed under reduced pressure and the crude reaction mixture was purified by flash column chromatography to give yellow solid (37 mg, 54%). ^{1}H NMR (400 MHz, Methanol-d_4) δ 9.18 (s, 1H), 8.29 (d, *J* = 5.6 Hz, 1H), 8.20 (s, 1H), 8.10 (d, *J* = 8.9 Hz, 1H), 7.82 (d, *J* = 6.3 Hz, 1H), 7.69 (dd, *J* = 9.0, 2.2 Hz, 1H), 4.15-3.89 (m, 3H), 3.82-3.63 (m, 4H), 2.54- 2.31 (m, 1H), 2.28-2.03 (m, 1H); LRMS (m/z): $[M+H]^+$ calcd. for $C_{16}H_{20}N_5O_2$, 314.2; found, 314.2.

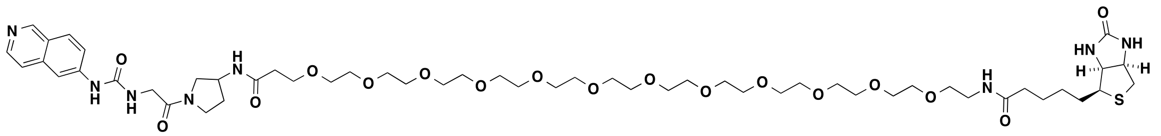

Synthesis of UNC3815: To the stirring mixture of above product (20.0 mg, 0.037 mmol) in DMF (0.3 mL) was added Biotin-PEG12-acid (28.3 mg, 0.034 mmol) followed by 1-[Bis(dimethylamino)methylene]-1H-1,2,3-triazolo[4,5-b]pyridinium 3-oxid hexafluorophosphate (HATU) (15.3 mg, 0.040 mmol) at room tempreture to give a clear yellow solution. To this solution was added diisopropylethylamine (DIPEA) (23 μL, 0.134 mmol) and resulting mixture was stirred at room temperature for 18 hours. The volatiles were removed under reduced pressure and the crude reaction mixture was purified by HPLC to give desired product as colorless oil (37.0 mg, 87%). ^{1}H NMR (400 MHz, Methanol-d_4) δ 9.44 (s, 1H), 8.48 (s, 1H), 8.38-8.33 (m, 2H), 8.19 (d, *J* = 6.7 Hz,

1H), 7.89 (dt, *J* = 9.0, 2.0 Hz, 1H), 4.52- 4.35 (m, 2H), 4.30 (dd, *J* = 7.9, 4.5 Hz, 1H), 4.17-4.00 (m, 2H), 3.83-3.43 (m, 52H), 3.36-3.33 (m, 2H), 3.23-3.18 (m, 1H), 2.93 (dd, *J* = 12.7, 5.0 Hz, 1H), 2.71 (d, *J* = 12.7 Hz, 1H), 2.49-2.43 (m, 2H), 2.32-2.13 (m, 3H), 2.09-1.93 (m, 1H), 1.80-1.53 (m, 4H), 1.47-1.39 (m, 2H);
HRMS (m/z): $[M+H]^+$ calcd. for $C_{53}H_{87}N_8O_{17}S$, 1139.5904; found, 1139.5897.

Procedure for the Preparation of UNC3811

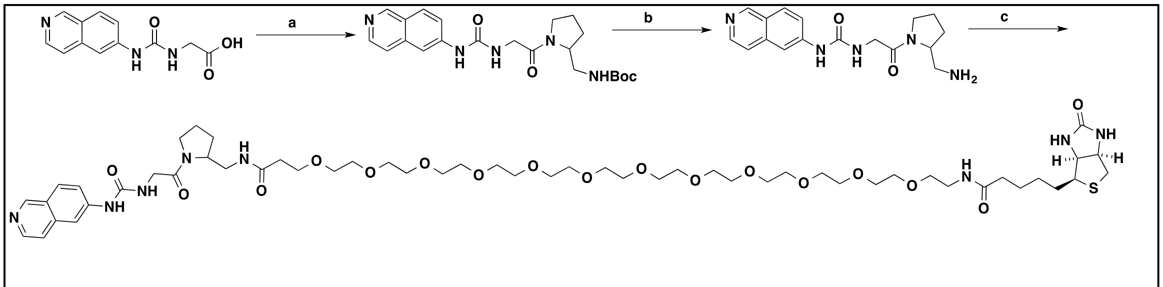

Reagents and conditions: (a) 2-Boc-(aminomethyl)pyrrolidine, EDC.HCl, HOAt, NMM, DMF, RT, 76%; (b) TFA, DCM, 0°C, 56% ; (c)Biotin- PEG-12-acid, HATU, DIPEA, DMF, RT, 93%

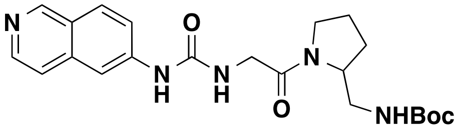

Synthesis of *tert*-butyl ((1-((isoquinolin-6-ylcarbamoyl)glycyl)pyrrolidin-2-yl)methyl)carbamate: To a stirring mixture of (isoquinolin-6-ylcarbamoyl)glycine (124 mg, 0.34 mmol, 1.0 eq) in DMF (3 mL) was added N-(3-Dimethylaminopropyl)-N′-ethylcarbodiimide hydrochloride (EDC.HCl) (131 mg, 0.69 mmol, 2.0 eq); 1-Hydroxy-7-azabenzotriazole (HOAt) (69 mg, 0.51 mmol, 1.5 eq) and 2-Boc-(aminomethyl)pyrrolidine (138 mg, 0.69 mmol, 2.0 eq) followed by N-Methylmorpholine (NMM) (76 µL, 0.69 mmol, 2.0 eq) and resulting mixture was stirred 18 hours at room temperature. The volatiles were removed under reduced pressure and the crude reaction mixture was purified by flash column chromatography to give white solid (124 mg, 84%). ^{1}H NMR (400 MHz, Methanol-d_4) δ 9.03 (s, 1H), 8.29 (d, *J* = 5.9 Hz, 1H), 8.09 (s, 1H), 7.97 (d, *J* = 8.9 Hz, 1H), 7.64 (d, *J* = 5.9 Hz, 1H), 7.59 (dd, *J* = 8.9, 2.0 Hz, 1H), 4.22-3.95 (m, 3H), 3.58-3.52 (m, 2H), 3.28-3.08 (m, 2H), 2.14-1.83 (m, 4H), 1.44 (br s, 9H);
LRMS (m/z): $[M+H]^+$ calcd. for C22H30N5O4, 428.2; found, 428.2.

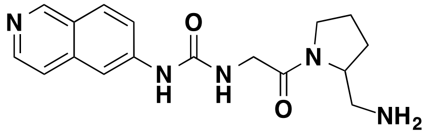

Synthesis of 1-(2-(2-(aminomethyl)pyrrolidin-1-yl)-2-oxoethyl)-3-(isoquinolin-6-yl)urea: To the stirring mixture of the above product (65.5 mg, 0.153 mmol, 1.0 eq) in dicholoromethane (1 mL) was added trifluoroacetic acid (TFA) (0.25 mL) at 0 °C and resulting mixture was stirred at 0 °C for 3.5 hrs. The volatiles were removed under reduced pressure and the crude reaction mixture was purified by flash column

chromatography to give off white solid (48 mg, 56%). ^{1}H NMR (400 MHz, Methanol-d_4) δ 9.01 (q, *J* = 0.9 Hz, 1H), 8.28 (d, *J* = 5.9 Hz, 1H), 8.07 (s, 1H), 7.96 (d, *J* = 8.9 Hz, 1H), 7.62 (d, *J* = 5.9 Hz, 1H), 7.57 (dd, *J* = 8.9, 2.0 Hz, 1H), 4.13-4.10 (m, 1H), 4.06 (br s, 2H), 3.59-3.53 (m, 2H), 2.86 (dd, *J* = 12.9, 4.9 Hz, 1H), 2.71 (dd, *J* = 12.9, 7.0 Hz, 1H), 2.10-1.84 (m, 4H).
LRMS (m/z): $[M+H]^+$ calcd. for $C_{17}H_{22}N_5O_2$, 328.2; found, 328.2.

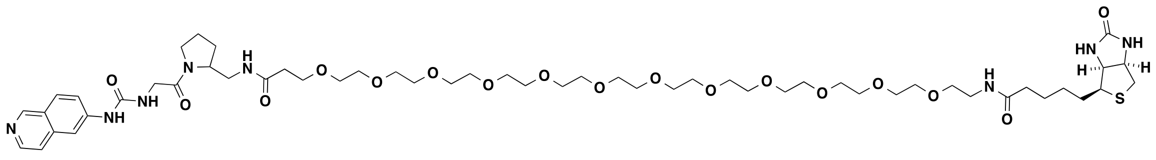

Synthesis of UNC3811: To the stirring mixture of above product (22.0 mg, 0.040 mmol) in DMF (0.3 mL) was added Biotin-PEG12-acid (30.0 mg, 0.036 mmol) followed by 1-[Bis(dimethylamino)methylene]-1H-1,2,3-triazolo[4,5-b]pyridinium 3-oxid hexafluorophosphate (HATU) (16.4 mg, 0.043 mmol) at room temperature to give a clear yellow solution. To this solution was added diisopropylethylamine (DIPEA) (25 μL, 0.144 mmol) and resulting mixture was stirred at room temperature for 18 hours. The volatiles were removed under reduced pressure and the crude reaction mixture was purified by HPLC to give desired product as colorless oil (45.3 mg, 93%). ^{1}H NMR (400 MHz, Methanol-d_4) δ 9.45 (d, *J* = 2.0 Hz, 1H), 8.48 (s, 1H), 8.40-8.32 (m, 2H), 8.19 (d, *J* = 7.7 Hz, 1H), 7.89 (dd, *J* = 9.0, 2.0 Hz, 1H), 4.53-4.47 (m, 1H), 4.32-4.16 (m, 2H), 4.08 (s, 2H), 3.76-3.52 (m, 52H), 3.36-3.33 (m, 2H), 3.23-3.16 (m, 1H), 2.92 (dd, *J* = 12.7, 5.0 Hz, 1H), 2.74-2.64 (m, 1H), 2.51-2.43 (m, 2H), 2.21 (t, *J* = 7.4 Hz, 2H), 2.16 -1.82 (m, 4H), 1.78-1.54 (m, 4H), 1.47-1.40 (m, 2H);
HRMS (m/z): $[M+H]^+$ calcd. for $C_{54}H_{89}N_8O_{17}S$, 1153.6061; found, 1153.6060.

Procedure for the preparation of SGC1442 and SGC0784

The procedure described for the synthesis of **16d** (Supplementary Information, *Journal of Medicinal Chemistry*, DOI: 10.1021/acs.jmedchem.5b01630) was adapted for the synthesis of (**Y**) and (**Z**), precursors for the synthesis of **SGC1442** and **SGC0784** respectively (see below). The only changes made involved replacement of 1-methylpiperazine with morpholine for the synthesis of (**Z**), and replacement of 4-(3-(4,4,5,5-tetramethyl-1,3,2-dioxaborolan-2-yl)benzyl)morpholine in General Procedure F with *tert*-butyl 4-(3-(4,4,5,5-tetramethyl-1,3,2-dioxaborolan-2-yl)benzyl)piperazine-1-carboxylate for the synthesis of both (**Y**) and (**Z**).

***N*-(3'-((4-(5,21-Dioxo-25-((3a*S*,4*S*,6a*R*)-2-oxohexahydro-1*H*-thieno[3,4-*d*]imidazol-4-yl)-10,13,16-trioxa-6,20-diazapentacosanoyl)piperazin-1-yl)methyl)-4-(4-methylpiperazin-1-yl)-[1,1'-biphenyl]-3-yl)-6-oxo-4-(trifluoromethyl)-1,6-dihydropyridine-3-carboxamide (SGC1442)**

_N_-(3'-((4-(5,21-dioxo-25-((3a_S_,4_S_,6a_R_)-2-oxohexahydro-1_H_-thieno[3,4-_d_]imidazol-4-yl)-10,13,16-trioxa-6,20-diazapentacosanoyl)piperazin-1-yl)methyl)-4-morpholino-[1,1'-biphenyl]-3-yl)-6-oxo-4-(trifluoromethyl)-1,6-dihydropyridine-3-carboxamide (SGC0784)

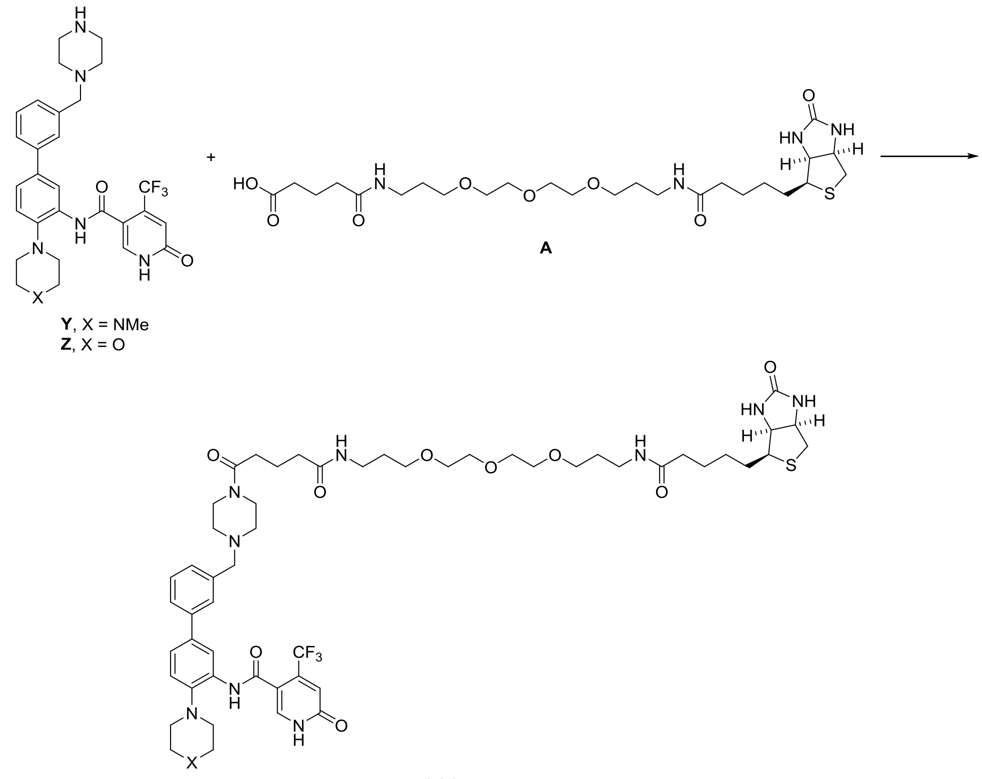

Y, X = NMe
Z, X = O

SGC1442, X = NMe
SGC0784, X = O

To a solution of either _N_-(4-(4-methylpiperazin-1-yl)-3'-(piperazin-1-ylmethyl)-[1,1'-biphenyl]-3-yl)-6-oxo-4-(trifluoromethyl)-1,6-dihydropyridine-3-carboxamide (**Y**) or _N_-(4-morpholino-3'-(piperazin-1-ylmethyl)-[1,1'-biphenyl]-3-yl)-6-oxo-4-(trifluoromethyl)-1,6-dihydropyridine-3-carboxamide (**Z**) (0.019 mmol) and 5,21-dioxo-25-((3a_S_,4_S_,6a_R_)-2-oxohexahydro-1_H_-thieno[3,4-_d_]imidazol-4-yl)-10,13,16-trioxa-6,20-diazapentacosanoic acid·DIPEA (**A**) (0.019 mmol) in DMF (0.5 mL) at room temperature was added HATU (0.019 mmol) and _N_,_N_-diisopropylethylamine (9.74 µL, 0.056 mmol). The resulting solution was stirred for 16 hours prior to removal of the solvent under reduced pressure. The residue was purified by prep HPLC (see below for conditions) followed by additional purification using a cation exchange column (PoraPak RxnCX 6 cc column, acidic PE resin, eluting with 3% NH_4OH in MeOH v/v) to afford the title compounds as white powders (>70% yield). **SGC1442** ^{1}H NMR (500 MHz, MeOD) δ 8.32 (s, 1H), 8.11 (s, 1H), 7.63 (s, 1H), 7.55 (s, 1H), 7.47 – 7.45 (m, 1H), 7.41 (t, _J_ = 7.6 Hz, 2H), 7.35 (s, 1H), 6.80 (s, 1H), 4.50 – 4.44 (m, 2H), 4.28 (dd, _J_ = 7.8, 4.5 Hz, 2H), 3.65 – 3.58 (m, 16H), 3.50 (d, _J_ = 1.1 Hz, 4H), 3.25 (s, 4H), 3.01 – 2.99 (m, 4H), 2.91 (dd, _J_ = 12.7, 4.9 Hz, 2H), 2.51 – 2.49 (m, 4H), 2.41 (d, _J_ = 7.5 Hz, 2H), 2.36 (s, 3H), 2.23 (t, _J_ = 7.2 Hz, 4H), 2.18 (t, _J_ = 7.3 Hz, 3H), 1.88 (dd, _J_ = 13.0, 5.3 Hz, 4H), 1.77 – 1.71 (m, 8H), 1.67 – 1.59 (m, 4H); ^{13}C NMR (151 MHz, MeOD) δ 175.92, 175.33, 173.26, 166.07, 165.99, 144.42, 143.83, 141.93, 139.16, 138.82, 137.67, 134.14, 129.94, 129.50, 129.45, 128.97, 126.05 (q, _J_ = 290.2 Hz), 122.04, 117.55, 114.94, 110.96, 71.53, 71.23, 69.92, 63.67, 63.37,

61.60, 57.01, 56.36, 54.16, 53.88, 52.69, 46.66, 46.10, 42.65, 41.04, 40.42, 37.82, 36.85, 36.22, 33.23, 30.43, 29.81, 29.51, 26.89, 22.72; LRMS (m/z): $[M+H]^+$ calcd. for $C_{54}H_{76}F_3N_{10}O_9S$, 1097.5; found, 1098.2.

SGC0784 ^{1}H NMR (500 MHz, MeOD) δ 8.30 (s, 1H), 8.07 (s, 1H), 7.55 (d, *J* = 8.0 Hz, 1H), 7.52 (d, *J* = 7.7 Hz, 1H), 7.47 (dd, *J* = 8.3, 1.9 Hz, 1H), 7.43 – 7.39 (m, 2H), 7.33 (s, 1H), 6.84 (s, 1H), 4.50 – 4.44 (m, 2H), 4.28 (dd, *J* = 7.7, 4.4 Hz, 2H), 3.91 – 3.88 (m, 3H), 3.86 – 3.83 (m, 3H), 3.62 (s, 16H), 3.51 (s, 4H), 3.25 (s, 4H), 2.94 – 2.92 (m, 4H), 2.69 (d, *J* = 12.9 Hz, 2H), 2.50 (d, *J* = 7.7 Hz, 4H), 2.41 (t, *J* = 7.5 Hz, 4H), 2.23 (s, 3H), 2.18 (t, *J* = 7.3 Hz, 4H), 1.75 (s, 5H), 1.63 (dd, *J* = 14.9, 7.5 Hz, 3H); ^{13}C NMR (151 MHz, MeOD) δ 175.93, 175.33, 173.26, 166.08, 165.79, 144.70, 144.21, 141.90, 139.17, 138.81, 138.47, 134.08, 129.95, 129.91, 129.52, 129.42, 128.97, 128.92, 127.01, 126.11 (q, *J* = 256.3 Hz), 124.70, 122.34, 122.22, 122.07, 121.61, 118.25, 114.87, 71.53, 71.23, 69.92, 68.30, 63.67, 63.37, 61.61, 57.01, 54.17, 53.86, 53.62, 53.48, 46.66, 42.66, 41.05, 40.42, 37.82, 36.85, 36.22, 33.24, 30.43, 29.81, 29.51, 26.89, 22.72; LRMS (m/z): $[M+H]^+$ calcd. for $C_{53}H_{73}F_3N_9O_{10}S$, 1084.5; found, 1085.2.

Agilent Prep HPLC

Column: XSelect Prep C18 5 μm, 10x100 mm

Flow: 5 mL/min

Run time: 15 min

A: MeCN

B: 10 mM Ammonium Bicarbonate (3.2 g/4 L)

Time	A	B
0	10%	90%
11	65%	35%
12	90%	10%
13	90%	10%
14	10%	90%
15	10%	90%

Procedure for the preparation of (A-395)-biotin.

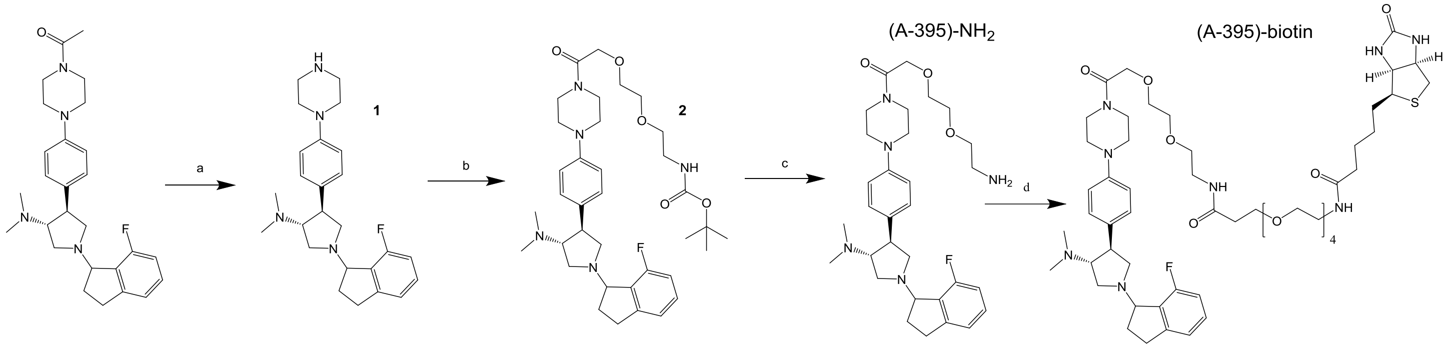

Reagents: (a) 6 M HCl, THF, CH_3OH, rt, 64%; (b) HOOC-dPEG$_2$-NHBoc, PyAOP, DIEA, DMF, rt, 51%; (c) TFA, rt; (d) NHS-dPEG$_4$-biotin, DIEA, DMSO, rt, 61%.

(3R,4S)-1-(7-fluoro-2,3-dihydro-1H-inden-1-yl)-N,N-dimethyl-4-(4-(piperazin-1-yl)phenyl)pyrrolidin-3-amine 1: 1-(4-(4-((3S,4R)-4-(dimethylamino)-1-(7-fluoro-2,3-dihydro-1H-inden-1-yl)pyrrolidin-3-yl)phenyl)piperazin-1-yl)ethanone (100 mg, 0.222 mmol) in THF (740 μL) and MeOH (740 μL) at 23 °C was treated with 6 M HCl (444 μL, 2.66 mmol) [8]. The clear solution was stirred overnight at 23 °C to partial conversion, then at 60°C for 7h. The solution was added to 20 mL sat. sodium bicarbonate and extracted with 2 × 20 mL ethyl acetate. The combined organic layers were washed with brine, dried over $MgSO_4$ and concentrated. The residue was chromatographed (Biotage 10g HP SNAP cartridge, methanol/dichloromethane gradient 5-20%) to afford **1** (58 mg, 64%) as a 60:40 mixture of epimers: ^{1}H NMR (400 MHz, DMSO-d_6) δ ppm 7.33 – 7.17 (m, 1H), 7.17 – 7.01 (m, 3H), 6.93 (td, *J* = 8.8, 5.2 Hz, 1H), 6.78 (dd, *J* = 8.7, 3.3 Hz, 2H), 4.30 (dd, *J* = 7.4, 1.8 Hz, 1H), 4.16 (dd, *J* = 7.0, 1.7 Hz, 1H), 3.25 – 2.92 (m, 6H), 2.92 – 2.71 (m, 8H), 2.63 (dd, *J* = 9.0, 5.7 Hz, 1H), 2.45 – 2.27 (m, 1H), 2.27 – 1.76 (m, 8H); LRMS (m/z): $[M+H]^+$ calcd. for $C_{25}H_{34}FN_4$, 409.3; found, 409.2.

***Tert*-butyl (2-(2-(2-(4-(4-((3S,4R)-4-(dimethylamino)-1-(7-fluoro-2,3-dihydro-1H-inden-1-yl)pyrrolidin-3-yl)phenyl)piperazin-1-yl)-2-oxoethoxy)ethoxy)ethyl)carbamate tris-trifluoroacetate 2**: (3R,4S)-1-(7-fluoro-2,3-dihydro-1H-inden-1-yl)-N,N-dimethyl-4-(4-(piperazin-1-yl)phenyl)pyrrolidin-3-amine (**1**) and 2,2-dimethyl-4-oxo-3,8,11-trioxa-5-azatridecan-13-oic acid (81 mg, 0.306 mmol) were combined in 2 mL anhydrous dimethylformamide. ((3H-[1,2,3]triazolo[4,5-b]pyridin-3-yl)oxy)tri(pyrrolidin-1-yl)phosphonium hexafluorophosphate(V) (191 mg, 0.367 mmol) and DIEA (N,N-diisopropylethylamine) (257 μL, 1.469 mmol) were added. The reaction was shaken at 23 °C. The reaction was diluted to 6 mL with 90% DMSO/water and purified in two injections by RP-HPLC on a Waters Deltapak C18 200 x 25 mm column eluted with a gradient of of 5% A (0.1% TFA-water):B (MeCN) to 95% A:B to give **2** (51.5 mg, 39.3%) as a white solid: LRMS (m/z): $[M+H]^+$ calcd. for $C_{36}H_{53}FN_5O_5$, 654.4; found, 654.3; $[M-H]^-$ calcd. for $C_{36}H_{51}FN_5O_5$, 652.4; found, 652.2.
Analytical LCMS TFA Method. Analytical LCMS was performed on a Thermo MSQ-Plus mass spectrometer

and Agilent 1100/1200 HPLC system running Xcalibur 2.0.7, Open-Access 1.4, and custom login software. The mass spectrometer was operated under APCI or ESI ionization conditions as noted in experimentals. The HPLC system comprised an Agilent Binary pump, degasser, column compartment, autosampler and diode-array detector, with a Polymer Labs ELS-2100 evaporative light-scattering detector. The column used was a Phenomenex Kinetex C8, 2.6 μm 100Å (2.1mm × 30mm), at a temperature of 65°C. A gradient of 5-100% acetonitrile (B) and 0.1% trifluoroacetic acid in water (A) was used, at a flow rate of 1.5 mL/min (0-0.05 min 5% A, 0.05-1.2 min 5-100% A, 1.2-1.4 min 100% A, 1.4-1.5 min 100-5% A. 0.25 min post-run delay) to give an elution time (R_t) of 0.73 min; LRMS (m/z): $[M+H]^+$ calcd. for $C_{36}H_{53}FN_5O_5$, 654.4; found, 654.2.

2-(2-(2-aminoethoxy)ethoxy)-1-(4-(4-((3S,4R)-4-(dimethylamino)-1-(7-fluoro-2,3-dihydro-1H-inden-1-yl)pyrrolidin-3-yl)phenyl)piperazin-1-yl)ethan-1-one (A-395)-NH2: *Tert*-butyl (2-(2-(2-(4-(4-((3S,4R)-4-(dimethylamino)-1-(7-fluoro-2,3-dihydro-1H-inden-1-yl)pyrrolidin-3-yl)phenyl)piperazin-1-yl)-2-oxoethoxy)ethoxy)ethyl)carbamate tris-trifluoroacetate (**2**) (22 mg, 0.023 mmol) was dissolved in 1 mL trifluoroacetic acid and immediately evaporated to dryness under a stream of dry nitrogen gas to give **(A-395)-NH2** as a colorless film: Analytical LCMS TFA Method R_t 0.60 min, LRMS (m/z): $[M+H]^+$ calcd. for $C_{31}H_{45}FN_5O_3$, 554.3; found, 554.1.

N-(2-(2-(2-(4-(4-((3R,4S)-1-benzyl-4-(dimethylamino)pyrrolidin-3-yl)phenyl)piperazin-1-yl)-2-oxoethoxy)ethoxy)ethyl)-1-(5-((3aS,4S,6aR)-2-oxohexahydro-1H-thieno[3,4-d]imidazol-4-yl)pentanamido)-3,6,9,12-tetraoxapentadecan-15-amide bis-trifluoroacetate (A-395)-biotin: The crude **(A-395)-NH2** was combined with NHS-dPEG$_4$-biotin (25 mg, 0.042 mmol, Quantabiodesign, Product #10200) in 1 mL anhydrous DMSO to which was added 30 μL DIEA. The mixture was shaken at 23 °C for 16 h, diluted to 3 mL with 90% DMSO/water and purified in one injection by RP-HPLC on a Waters Deltapak C18 200 x 25 mm column eluted with a gradient of 5% A (0.1% TFA-water):B (MeCN) to 95% A:B to give **(A-395)-biotin** (15.3 mg, 60.7%) as a waxy solid: ^{1}H NMR (500 MHz, DMSO-d$_6$) δ ppm 7.92 (t, J = 5.6 Hz, 1H), 7.84 (t, J = 5.7 Hz, 1H), 7.40 (tdd, J = 8.2, 5.3, 3.5 Hz, 1H), 7.33 (d, J = 8.4 Hz, 2H), 7.20 (dd, J = 7.4, 2.0 Hz, 1H), 7.07 (dt, J = 17.8, 8.9 Hz, 1H), 6.97 (dd, J = 8.9, 2.4 Hz, 2H), 6.44 6.40 (m, 3H), 4.31 (dd, J = 7.8, 4.9 Hz, 1H), 4.22 4.10 (m, 4H), 3.63 3.52 (m, 9H), 3.50 (s, 6H), 3.55 3.43 (m, 7H), 3.40 (dt, J = 12.0, 5.9 Hz, 4H), 3.19 (dq, J = 8.7, 5.9 Hz, 4H), 3.15 (s, 5H), 3.15 3.06 (m, 2H), 2.94 2.78 (m, 2H), 2.72 (d, J = 11.0 Hz, 6H), 2.58 (d, J = 12.5 Hz, 1H), 2.32 (t, J = 6.5 Hz, 2H), 2.10 2.03 (m, 2H), 1.67 1.56 (m, 1H), 1.56 1.40 (m, 2H), 1.48 (s, 1H), 1.36 1.22 (m, 2H); ^{13}C NMR (125 MHz, DMSO-d$_6$) δ_C 172.14, 170.13, 167.29, 162.68, 159.0, 149.97, 149.37, 131.72, 129.12, 128.27, 124.59, 121.2, 115.84, 113.32, 69.7, 69.58, 69.53, 69.46, 69.34, 69.07, 69.01, 66.79, 68.6, 64.15, 61.02, 59.15, 55.34, 48.2, 43.11, 40.91, 40.74, 40.59, 39.71, 38.5, 38.43, 36.01, 35.05, 30.38, 29.03, 28.34, 28.06, 25.23; Analytical LCMS TFA Method R_t 0.71 min; LRMS (m/z): $[M+2H]^{2+}$ calcd. for $C_{52}H_{81}FN_8O_{10}S$, 514.3; found, 514.6; $[M+H]^+$ calcd. for $C_{52}H_{80}FN_8O_{10}S$, 1027.6; found, 1027.7; $[M-H]^-$ calcd. for $C_{52}H_{78}FN_8O_{10}S$, 1025.6; found, 1026.1.

Procedure for the preparation of (A-395N)-biotin

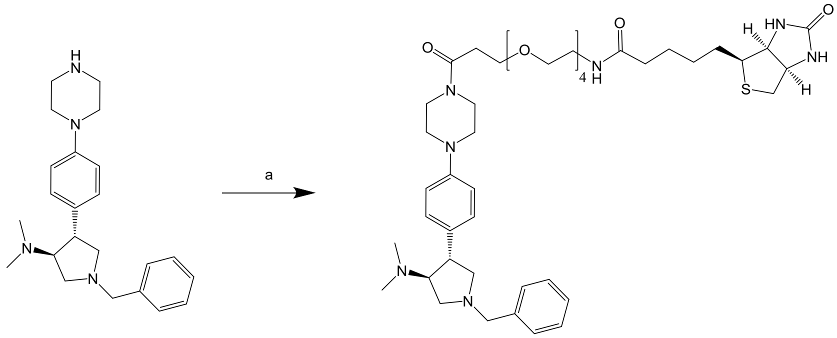

Reagents: (a) NHS-dPEG$_4$-biotin, DIEA, DMSO, rt, 98%.

N-(15-(4-(4-((3R,4S)-1-benzyl-4-(dimethylamino)pyrrolidin-3-yl)phenyl)piperazin-1-yl)-15-oxo-3,6,9,12-tetraoxapentadecyl)-5-((3aS,4S,6aR)-2-oxohexahydro-1H-thieno[3,4-d]imidazol-4-yl)pentanamide bis-trifluoroacetate (A-395N)-biotin: (3S,4R)-1-benzyl-N,N-dimethyl-4-(4-(piperazin-1-yl)phenyl)pyrrolidin-3-amine tris-trifluoroacetate (100 mg, 0.142 mmol) was combined with NHS-dPEG$_4$-biotin (50 mg, 0.085 mmol) in 1 mL anhydrous DMSO. N,N-diisopropylethylamine) (74.2 μL, 0.425 mmol) was added. The reaction was shaken for 16 h, diluted to 3 mL with 90% DMSO/water and purified in one injection by RP-HPLC on a Waters Deltapak C18 200 x 25 mm column eluted with a gradient of of 5% A (0.1% TFA-water):B (MeCN) to 95% A:B to give **(A-395N)-biotin** (90 mg, 98.9%) as a waxy solid: ^{1}H NMR (500 MHz, DMSO-*d*$_6$) δ ppm 7.84 (t, *J* = 5.7 Hz, 1H), 7.65 – 7.39 (m, 7H), 7.37 (d, *J* = 8.5 Hz, 2H), 7.10 – 6.88 (m, 2H), 6.43 (s, 2H), 4.31 (dd, *J* = 7.8, 4.9 Hz, 4H), 4.28 (s, 1H), 3.72 (m, 1H), 3.62 (dt, *J* = 24.5, 5.2 Hz, 4H), 3.62 – 3.58 (m, 4H), 3.52 – 3.48 (m, 12H), 3.39 (t, *J* = 11.9 Hz, 2H), 3.22 – 2.91 (m, 8H), 2.82 (dd, *J* = 12.4, 5.1 Hz, 1H), 2.72 (s, 6H), 2.67 – 2.50 (m, 3H), 2.07 (t, *J* = 7.4 Hz, 2H), 1.62 (m, 1H), 1.56 – 1.37 (m, 3H), 1.30 (m, 3H); ^{13}C NMR (125 MHz, DMSO-d$_6$) δ_C 172.1, 168.8, 162.68, 150.2, 130.04, 128.99, 128.81, 128.63, 127.65, 115.87, 69.66, 69.04, 67.85, 66.69, 60.97, 59.33, 59.16, 57.63, 55.37, 53.2, 48.34, 47.99, 43.02, 40.98, 40.68, 39.76, 38.38, 35.08, 32.75, 28.15, 28.0, 25.22; Analytical LCMS TFA Method R$_t$ 0.72 min; LRMS (m/z): [M+H]$^+$ calcd. for $C_{44}H_{68}FN_7O_7S$, 838.5; found, 838.5; [M+2H]$^{2+}$ calcd. for $C_{44}H_{69}FN_7O_7S$, 419.7; found, 420.0; [M-H]$^-$ calcd. for $C_{44}H_{66}FN_7O_7S$, 836.5; found, 835.7.

Procedure for the preparation of SGC3185.

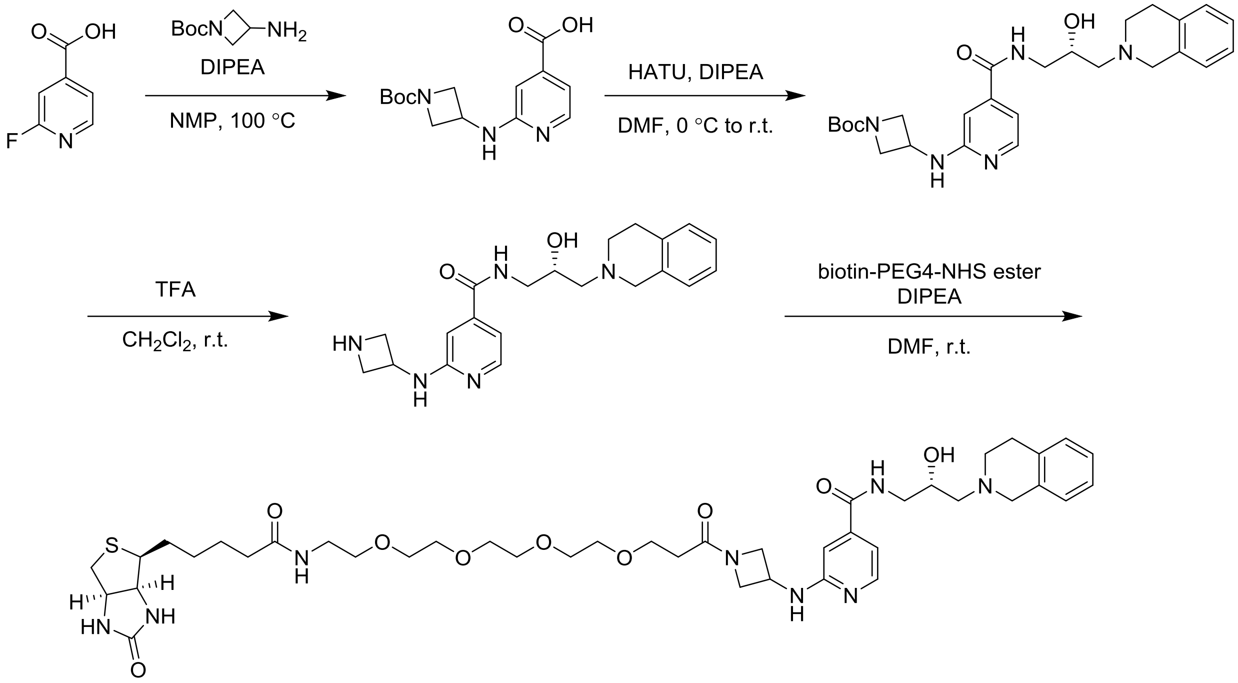

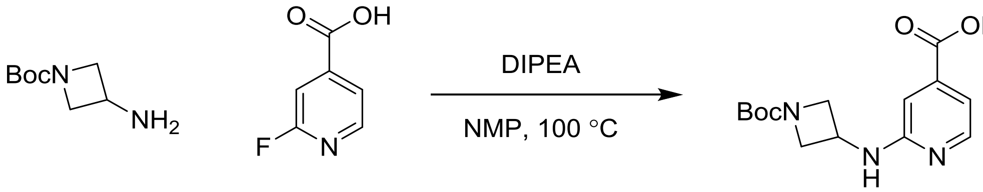

To 1-Boc-3-(amino)azetidine (305 mg, 1.772 mmol) and 2-Fluoro-4-pyridinecarboxylic acid (50 mg, 0.354 mmol) in N-methyl-2-pyrrolidinone (1 mL) was added N,N-Diisopropylethylamine (0.185 ml, 1.063 mmol) and the reaction mixture heated to 100 °C for 42 h. All volatiles were then evaporated and the crude reaction mixture dissolved in water and column chromatographed (RP-C18, H_2O (0.1% FA v/v)/MeCN (0.1% FA v/v), 95:5 to 30:70 v/v) to afford 2-((1-(tert-butoxycarbonyl)azetidin-3-yl)amino)isonicotinic acid (60.4 mg, 58 % yield) as a white solid, which was used immediately. HPLC R_t 1.3 min. 99% pure by UV_{254}; 1H NMR (500 MHz, MeOD) δ 8.07 (d, *J* = 5.7 Hz, 1H), 7.32 – 7.21 (m, 3H), 7.18 (d, *J* = 7.3 Hz, 1H), 6.95 (s, 2H), 4.61 – 4.52 (m, 1H), 4.48 (s, 2H), 4.35 (s, 1H), 4.28 (t, *J* = 7.9 Hz, 2H), 3.85 – 3.74 (m, 2H), 3.62 (t, *J* = 5.9 Hz, 2H), 3.53 (dd, *J* = 13.6, 4.4 Hz, 1H), 3.47 (dd, *J* = 13.6, 5.3 Hz, 1H), 3.35 (d, *J* = 12.6 Hz, 1H), 3.29 – 3.12 (m, 3H), 1.45 (s, 9H); LRMS (m/z): $[M+H]^+$ calcd. for $C_{14}H_{20}N_3O_4$, 294.1; found, 294.4.

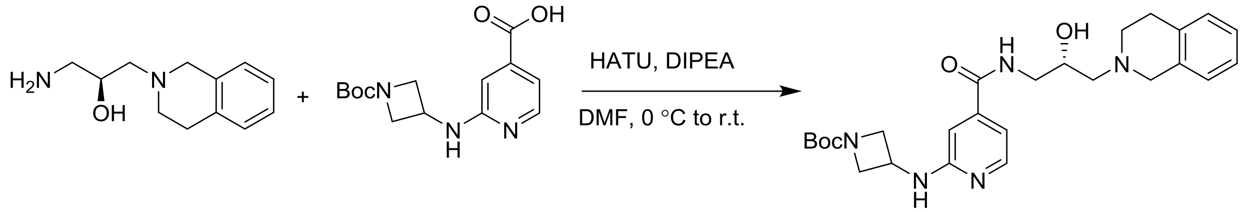

To 2-((1-(tert-butoxycarbonyl)azetidin-3-yl)amino)isonicotinic acid (60.4 mg, 0.206 mmol) and (S)-1-amino-3-(3,4-dihydroisoquinolin-2(1H)-yl)propan-2-ol (54.6 mg, 0.196 mmol) in DMF (5 mL) at 0 °C were added DIPEA (0.14 mL, 0.824 mmol) and HATU (95 mg, 0.247 mmol). The reaction mixture was stirred at this temperature for an hour and then allowed to warm slowly to room temperature overnight. All volatiles were then evaporated and the crude reaction mixture column chromatographed (RP-C18, H_2O (0.1% FA v/v)/MeCN, 98:2 to 10:90 v/v) to afford tert-butyl (S)-3-((4-((3-(3,4-dihydroisoquinolin-2(1H)-yl)-2-hydroxypropyl)carbamoyl)pyridin-2-yl)amino)azetidine-1-carboxylate (57.6 mg, 61 % yield) as a yellow solid. ^{1}H NMR (500 MHz, MeOD) δ 8.07 (d, *J* = 5.7 Hz, 1H), 7.32 – 7.21 (m, 3H), 7.18 (d, *J* = 7.3 Hz, 1H), 6.95 (s, 2H), 4.61 – 4.52 (m, 1H), 4.48 (s, 2H), 4.35 (s, 1H), 4.28 (t, *J* = 7.9 Hz, 2H), 3.85 – 3.74 (m, 2H), 3.62 (t, *J* = 5.9 Hz, 2H), 3.53 (dd, *J* = 13.6, 4.4 Hz, 1H), 3.47 (dd, *J* = 13.6, 5.3 Hz, 1H), 3.35 (d, *J* = 12.6 Hz, 1H), 3.29 – 3.12 (m, 3H), 1.45 (s, 9H); LCMS R_t 1.28 min, $[M+1]^+$; Purity (UV_{254}) 99%; LRMS (m/z): $[M+H]^+$ calcd. for $C_{26}H_{36}N_5O_4$, 482.3; found, 482.7.

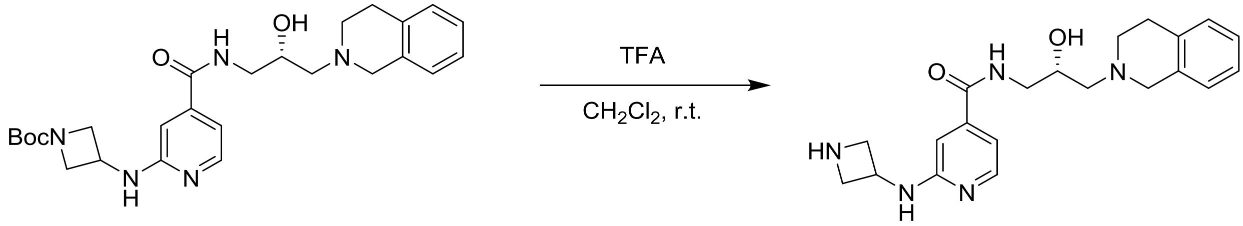

To tert-butyl (S)-3-((4-((3-(3,4-dihydroisoquinolin-2(1H)-yl)-2-hydroxypropyl)carbamoyl)pyridin-2-yl)amino)azetidine-1-carboxylate (57.6 mg, 0.120 mmol) in CH_2Cl_2 (10 mL) was added TFA (0.5 mL) at room temperature. After stirring overnight, the reaction mixture was concentrated under reduced pressure and the residue dissolved in MeOH (2 mL) and passed through a Biotage SCX-2 cation exchange column washing with MeOH (10 mL) and eluting with NH_4OH (3% w/v in MeOH, 10 mL) to afford (S)-2-(azetidin-3-ylamino)-N-(3-(3,4-dihydroisoquinolin-2(1H)-yl)-2-hydroxypropyl)isonicotinamide (41.5 mg, 91 % yield) as a yellow powder following lyophilisation from MeCN/H_2O. LRMS (m/z): $[M+H]^+$ calcd. for $C_{21}H_{28}N_5O_2$, 382.2; found, 382.7. Crude product used in the next step without purification.

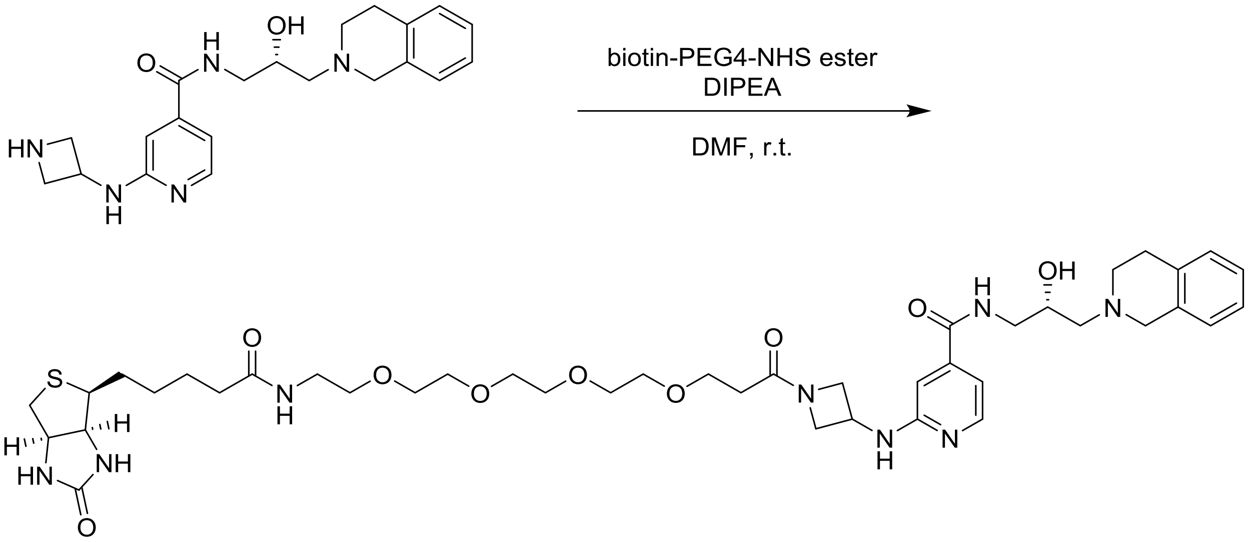

Synthesis of DAM-0010-0593-10: To biotin-PEG4-NHS ester (35.3 mg, 0.060 mmol) in DMF (0.6 mL) at 0 °C were added DIPEA (15 µL, 0.090 mmol) and (S)-2-(azetidin-3-ylamino)-N-(3-(3,4-dihydroisoquinolin-2(1H)-yl)-2-hydroxypropyl)isonicotinamide (22.9 mg, 0.060 mmol) in DMF (0.5 mL). The solution was allowed to slowly warm to room temperature and stirred overnight. The crude reaction mixture was then purified by preparative HPLC (see conditions below) to afford N-((S)-3-(3,4-dihydroisoquinolin-2(1H)-yl)-2-hydroxypropyl)-2-((1-(17-oxo-21-((3aS,4S,6aR)-2-oxohexahydro-1H-thieno[3,4-d]imidazol-4-yl)-4,7,10,13-tetraoxa-16-azahenicosanoyl)azetidin-3-yl)amino)isonicotinamide formic acid salt (11.1 mg, 21.96 % yield) as a white solid following lyophilisation from MeCN/H_2O.

Prep purification Waters instrument:

Column: XSelect Prep C18-HSS 5 µm, 10X100 mm

Flow: 8.0 mL/min

Run time: 15 min

Retention time: ~5 min

A2: MeCN (formic acid, 0.1% v/v)

B2: H_2O (formic acid, 0.1% v/v)

time	A2	B2
0	5%	95%
9	40%	60%
12	90%	10%
13	90%	10%
14	5%	95%
15	5%	95%

^{1}H NMR (500 MHz, MeOD) δ 8.10 (d, J = 5.3 Hz, 1H), 7.33 – 7.23 (m, 3H), 7.19 (d, J = 7.5 Hz, 1H), 6.95 (d, J = 5.5 Hz, 2H), 4.65 – 4.59 (m, 2H), 4.51 – 4.44 (m, 3H), 4.39 – 4.32 (m, 2H), 4.29 (dd, J = 7.9, 4.5 Hz, 1H), 4.09 (q, J = 8.7 Hz, 1H), 3.85 (dd, J = 10.4, 3.8 Hz, 1H), 3.74 (t, J = 6.1 Hz, 2H), 3.66 – 3.58 (m, 14H), 3.57 – 3.51 (m, 3H), 3.46 (dd, J = 13.7, 4.7 Hz, 1H), 3.36 – 3.33 (m, 3H), 3.25 (d, J = 10.3 Hz, 1H), 3.23 – 3.15 (m, 3H), 2.92 (dd, J = 12.8, 4.9 Hz, 1H), 2.70 (d, J = 12.7 Hz, 1H), 2.42 (t, J = 6.0 Hz, 2H), 2.20 (t, J = 7.4 Hz, 2H), 1.76 – 1.54 (m, 4H), 1.47 – 1.39 (m, 2H); LCMS HSS R_t 1.24 min; Purity (UV_{254}) 99%; LRMS (m/z): $[M+H]^+$ calcd. for $C_{42}H_{63}N_8O_9S$, 855.4; found, 855.7.

References

1. Liu F, *et al.* Discovery of an in vivo chemical probe of the lysine methyltransferases G9a and GLP. *J Med Chem* **56**, 8931-8942 (2013).

2. Pappano WN, *et al.* The Histone Methyltransferase Inhibitor A-366 Uncovers a Role for G9a/GLP in the Epigenetics of Leukemia. *PLoS One* **10**, e0131716 (2015).

3. He Y, *et al.* The EED protein-protein interaction inhibitor A-395 inactivates the PRC2 complex. *Nat Chem Biol* **13**, 389-395 (2017).

4. Konze KD, *et al.* An orally bioavailable chemical probe of the Lysine Methyltransferases EZH2 and EZH1. *ACS Chem Biol* **8**, 1324-1334 (2013).

5. Eggert E, *et al.* Discovery and Characterization of a Highly Potent and Selective Aminopyrazoline-Based in Vivo Probe (BAY-598) for the Protein Lysine Methyltransferase SMYD2. *J Med Chem* **59**, 4578-4600 (2016).

6. Gilan O, *et al.* Functional interdependence of BRD4 and DOT1L in MLL leukemia. *Nat Struct Mol Biol* **23**, 673-681 (2016).

7. Bonday ZQ, *et al.* LLY-283, a potent and selective inhibitor of arginine methyltransferase 5, PRMT5, with antitumor activity. *ACS Med Chem Lett,* (2018).

8. Curtin ML, *et al.* SAR of amino pyrrolidines as potent and novel protein-protein interaction inhibitors of the PRC2 complex through EED binding. *Bioorg Med Chem Lett* **27**, 1576-1583 (2017).
