# Supplementary material for: A chemical biology toolbox to study protein methyltransferases and epigenetic signaling
Source: Nat Commun. 2019 Jan 3;10:19. doi: 10.1038/s41467-018-07905-4 (PMC6318333; doi:10.1038/s41467-018-07905-4)
Supplement: Supplementary file 2 — Description of Additional Supplementary Files [file 41467_2018_7905_MOESM2_ESM.pdf]

Description of Additional Supplementary Files

1. File Name: Supplementary Data 1_PRMTi.xlsx
   Description: Label-free quantification of MS023 interactors in HEK293 cell lysate

2. File Name: Supplementary Data 2_EED.xlsx
   Description: Label-free quantification of A395 interactors in G401 cell lysate

3. File Name: Supplementary Data 3_DOT1L.xlsx
   Description: Label-free quantification of SGC0946 interactors in Jurkat cell lysate
